# PRMT5-mediated regulation of developmental myelination

**DOI:** 10.1038/s41467-018-04863-9

**Published:** 2018-07-19

**Authors:** Antonella Scaglione, Julia Patzig, Jialiang Liang, Rebecca Frawley, Jabez Bok, Angeliki Mela, Camila Yattah, Jingxian Zhang, Shun Xie Teo, Ting Zhou, Shuibing Chen, Emily Bernstein, Peter Canoll, Ernesto Guccione, Patrizia Casaccia

**Affiliations:** 1 Neuroscience Initiative at the Advanced Science Research Center of the Graduate Center of The City University of New York, 85 St. Nicholas Terrace, New York, NY 10031 USA; 2 Department of Neuroscience, Icahn School of Medicine at Mount Sinai, 1 Gustave L. Levy Pl, New York, NY 10029 USA; 3 Graduate School of Biomedical Sciences, Icahn School of Medicine at Mount Sinai, 1 Gustave L. Levy Pl, New York, NY 10029 USA; 4 Institute of Molecular and Cell Biology (IMCB), A*STAR (Agency for Science, Technology and Research), 61 Biopolis Drive, Proteos Building #3-06, Singapore, 138673 Singapore; 5 Department of Pathology and Cell Biology, Columbia University Medical Center, 630 West 168th Street, New York, NY 10032 USA; 6 Graduate Program in Biochemistry, The Graduate Center of The City University of New York, 365 5th Avenue, New York, NY 10016 USA; 7 Room A-829, Weill Cornell Medical College, 1300 York Avenue, New York, NY 10065 USA; 8 Department of Oncological Sciences, Icahn School of Medicine at Mount Sinai, 1425 Madison Avenue, New York, NY 10029 USA

## Abstract

Oligodendrocytes (OLs) are the myelin-forming cells of the central nervous system. They are derived from differentiation of oligodendrocyte progenitors through a process requiring cell cycle exit and histone modifications. Here we identify the histone arginine methyl-transferase PRMT5, a molecule catalyzing symmetric methylation of histone H4R3, as critical for developmental myelination. PRMT5 pharmacological inhibition, CRISPR/cas9 targeting, or genetic ablation decrease p53-dependent survival and impair differentiation without affecting proliferation. Conditional ablation of *Prmt5* in progenitors results in hypomyelination, reduced survival and differentiation. Decreased histone H4R3 symmetric methylation is followed by increased nuclear acetylation of H4K5, and is rescued by pharmacological inhibition of histone acetyltransferases. Data obtained using purified histones further validate the results obtained in mice and in cultured oligodendrocyte progenitors. Together, these results identify PRMT5 as critical for oligodendrocyte differentiation and developmental myelination by modulating the cross-talk between histone arginine methylation and lysine acetylation.

## Abstract (web summary)

Myelin-forming cells derive from oligodendrocyte progenitors. Here the authors identify histone arginine methyl-transferase PRMT5 as critical for developmental myelination by modulating the cross-talk between histone arginine methylation and lysine acetylation, to favor differentiation.

## Introduction

Brain function is highly specialized and dependent on the integrated action of several cell types. Oligodendrocytes (OLs) are the myelin-forming cells of the central nervous system (CNS) and are responsible for ensuring axonal conduction and neuronal support^1^. Their number is tightly regulated and dependent on differentiation, survival, and proliferation of oligodendrocyte progenitor cells (OPCs). Therefore, understanding the basic processes regulating OL cell number is key for the advancement in neurobiology.

We and others have previously contributed to elucidating the molecular mechanisms governing proliferation and differentiation of OPC^2–4^. Among the latter, we reported decreased acetylation of lysine residues on histone tails as an essential event for the differentiation of OPCs into OL^2,5–9^. Based on these and additional studies^10,11^, we proposed a mechanism of developmental myelination driven by de-repression of inhibitory molecules^9,12^.

Besides modifications of lysine residues, repressive modifications of nucleosomal histones include the symmetric dimethylation of arginines (ω-NG, ω-N′G-dimethyl arginine), which is mediated by class-II protein arginine methyltransferases (PRMTs) such as PRMT5^13,14^ and PRMT9^15,16^. PRMT5 is expressed in the brain and enriched in the OL lineage^17–19^. Its activity is thought to negatively regulate gene expression due to methylation of multiple arginine residues on nucleosomal histone tails^20–22^. PRMT5 is also expressed at high levels in proneural gliomas, which are transcriptionally related to OPCs^23,24^, and arise from their transformation^25,26^. PRMT5 levels positively correlate with malignancy and negatively correlate with glioma patients’ survival^27,28^, therefore justifying the efforts to identify specific pharmacological inhibitors as potential therapeutic targets^27,29–33^. Despite several studies highlighting the importance of PRMT5 in malignancies, the physiological role of this enzyme in the OL lineage remains poorly understood.

Previous studies in neural stem cells underlined the importance of PRMT5 in the regulation of pre-mRNA splicing^34^. Another study in a glial cell line suggested this enzyme could affect OL differentiation by affecting transcription, although the mechanistic aspects were not elucidated^19^. Based on this cumulative evidence, we reasoned that a thorough characterization of PRMT5 in the OL lineage is timely and may shed some light on a better understanding of the regulation of OL cell number in the brain. In this study, we adopted several strategies to address this key question including: a detailed characterization of mice with cell-lineage-specific ablation of *Prmt5* in immature oligodendrocyte progenitors or in oligodendrocytes, the use of CRISPR/Cas9 and pharmacological inhibitors to interfere with PRMT5 function in primary OPC cultures, transcriptomic analyses, and biochemical assays using synthetic proteins and modified histone peptides.

Because the study of symmetric arginine methylation relies on the high quality of reagents, in this study we extensively characterized the specificity of all the commercially available antibodies to study this modification and selected those with the highest level of discriminatory power from other modifications (including asymmetric methylation at the same residue). Overall, this comprehensive study identifies PRMT5 as a key regulator of the number of myelinating cells in the CNS, by modulating survival of differentiating progenitors and orchestrating a tight coordination between symmetric histone arginine methylation and decreased histone lysine acetylation at the transition between growth arrest and differentiation.

## Results

### PRMT5 expression and activity in the oligodendrocyte lineage

**Fig. 1:**
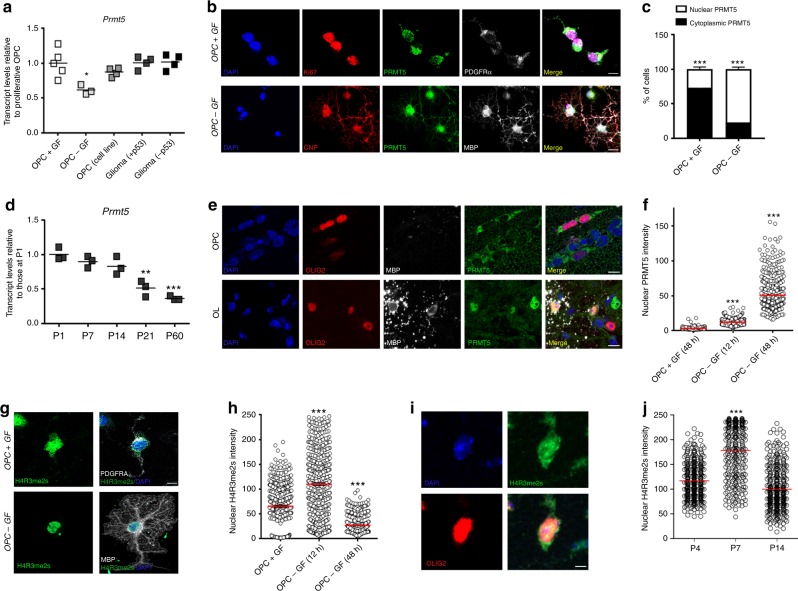
Subcellular localization of PRMT5 and histone H4R3me2s during oligodendrocyte differentiation. **a** *Prmt5* transcripts in proliferating (+GF), arrested OPC (−GF), OliNeu, or glioma cells. Scatter plots represent the average of three biological replicates, each in triplicate and normalized by the average of three housekeeping genes (*18s*, *Wdr33*, and *Pja2*). Values are referred as relative to those measured in OPC + GF (one-way ANOVA with Bonferroni’s multiple comparison test, *p < 0.05). **b** Confocal images of cells stained for PRMT5 (green), DAPI (blue), and either KI67 (red) and PDGFRα (white) in proliferating OPCs, or CNP (red) and MBP (white) in differentiating OPCs. Scale bar: 10 μm. **c** Relative quantification of cytoplasmic or nuclear PRMT5 in 150 PDGFRα^+^ or CNP^+^ cells. Values represent means ± sem from four biological replicates (one-way ANOVA, ***p < 0.001). **d** *Prmt5* transcripts in three biological RNA preparations from mouse spinal cord at indicated time points, normalized as described in **a** and referred to levels detected at P1 (one-way ANOVA with Bonferroni’s multiple comparison test, **p < 0.01, ***p < 0.001). **e** Confocal images of P7 brains stained for PRMT5 (green), OLIG2 (red), MBP (white), and DAPI (blue). Scale bar: 10 μM. **f** Quantification of nuclear PRMT5 intensity in OPCs. Scatter plots representing the average pixel intensity of 600 (for OPC + GF and OPC − GF12 h) and 569 (OPC − GF 48 h) nuclei quantified per condition (technical triplicates of four different biological preparations). One-way ANOVA, ***p < 0.001. **g** Confocal image of the PRMT5-specific mark H4R3me2s (green) in PDGFRα^+^ (white) or MBP^+^ (white) cells. DAPI (blue) as nuclear counterstaining. Scale bar: 5 μm. **h** Scatter plots representing the average pixel intensity of H4R3me2s-stained nuclei. Quantification of 468 nuclei of cells in proliferating conditions, 543 nuclei in growth-arrested, and 493 in differentiation conditions, each from four different preparations. One-way ANOVA, ***p < 0.001. **i** Representative confocal image of P4 mouse brain stained for H4Rme2s (green), OLIG2 (red), and DAPI (blue). Scale bar: 5 μm. **j** Scatter plots representing the average pixel intensity of H4R3me2s-stained nuclei of OLIG2^+^ at the indicated time points. Two hundred nuclei were quantified at each time point (50 cells/animal and four mice per time point. One-way ANOVA, ***p < 0.001

To characterize the expression pattern of *Prmt5* in OL lineage cells, we measured its transcript levels in RNA samples obtained from cultured primary oligodendrocyte progenitors (OPCs) kept either in proliferating or differentiating conditions, and compared with values from the immortalized OliNeu cell line or primary glioma cells. High levels of *Prmt5* were detected in proliferating OPCs, OliNeu, and glioma cells and lower transcripts in differentiating OPCs (Fig. 1a). At a subcellular level PRMT5 was found in the cytosol of proliferating OPCs (Fig. 1b) and in the nucleus of differentiating cells, after growth arrest induced by the withdrawal of growth factors (Fig. 1c, f).  *Prmt5* transcripts were high in the developing spinal cord at postnatal day 1 and then progressively declined over time (Fig. 1d). In the mouse brain its protein distribution was mostly cytoplasmic in OLIG2^+^ OPCs and nuclear in MBP^+^ differentiating OLs (Fig. 1e). This nucleo-cytoplasmic pattern was reminiscent of the dynamic changes in subcellular localization previously described for cell cycle regulators at the time of growth arrest^35^ and suggested that PRMT5 subcellular distribution is dependent on the proliferative state of the cells.

Because PRMT5 enzymatic activity is responsible for the symmetric methylation of arginine (R) residues on histone tails^36^, it is important to identify antibodies with the ability to discern this post-translational modification (PTM) in a very selective and specific manner (i.e., ascertain that other modifications of amino acid residues on the histone tails do not interfere with the specificity of epitope recognition by antibodies^37^). For this reason, prior to characterizing the enzymatic marks placed by PRMT5 in OL lineage cells, we conducted a systematic analysis of all the commercially available antibodies directed towards recognition of methylated arginine residues on histone tails (see Methods), using a modified Histone Peptide Array (Active Motif) (Supplementary Table 1; Supplementary Fig. 1). The assay included histone peptides with 384 permutations, including single or multiple combinations of PTMs on specific amino acid residues (a comprehensive list of all the PTM modifications can be accessed by using the link: https://www.activemotif.com/catalog/668/modified-histone-peptide-array and downloading the excel file). To study H4R3me2s, we selected the antibody that was able to recognize the methylated R epitope, regardless of the presence of PTM on adjacent lysine residues (Epigentek, Supplementary  Fig. 1). Antibody specificity was further assessed by the loss of signal in cells with CRISPR/Cas9 targeting of *Prmt5* (Supplementary Fig. 2).

Using these highly specific antibodies, we detected the PRMT5-dependent H4R3me2s histones in the cytoplasm of proliferating OPCs and in the nucleus of differentiating oligodendrocyte lineage cells, both  in culture (Fig. 1g, h) and in vivo (Fig. 1i, j). Importantly, the accumulation of nuclear H4R3me2s in primary cultured OPCs was detected at time of growth arrest^38,39^ after 12 h from growth factor withdrawal.

### Ablation of *Prmt5* results in severe hypomyelination

**Fig. 2:**
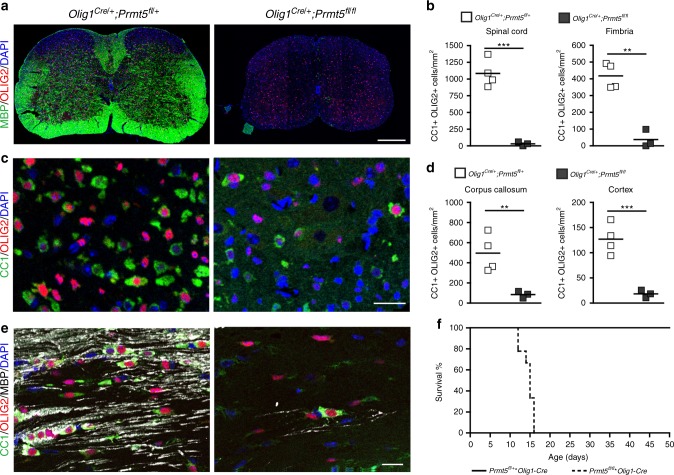
Genetic ablation of *Prmt5* in OPCs induces severe hypomyelination and impaired OPC differentiation. Confocal images of P14 spinal cord (**a**) and corpus callosum (**c**, **e**) sections from controls *(Olig1*^*Cre/+*^;*Prmt5*^*fl/+*^) and *Prmt5* mutants (*Olig1*^*Cre/+*^;*Prmt5*^*fl/fl*^) stained for MBP (**a**: green, scale bar = 200 μm; **e**: white, scale bar = 20 μm), CC1 (**c**, **e**: green, scale bar = 20 μm), OLIG2 (**a**: red, scale bar: 200 μm; **c**, **e**: red, scale bar = 20 μm). DAPI (blue) as nuclear counterstain. (**b**, **d**) Scatter plot represents the average number of CC1^+^/OLIG2^+^ cells quantified in spinal cord and fimbria (**b**) and in corpus callosum and cortex (**d**) of four control mice (*Olig1*^*Cre/+*^;*Prmt5*^*fl/+*^) and three *Prmt5* mutants (*Olig1*^*Cre/+*^;*Prmt5*^*fl/fl*^). Student’s *t* test, **p < 0.01, ***p < 0.001. **f** Survival curve showing comparison in controls (*Olig1*^*Cre/+*^;*Prmt5*^*fl/+*^) and nine *Prmt5* mutants (*Olig1*^*Cre/+*^;*Prmt5*^*fl/f*l^)

In order to test the functional importance of symmetric histone arginine methylation for myelination, we conditionally ablated *Prmt5* in OPCs, by crossing the *Prmt5*^*fl/fl*^ line with the OPC-specific *Olig1*-*Cre* driver line. Immunophenotypic characterization of these mice was performed by conducting confocal analysis of brain sections, stained with antibodies specific for the pan-OL lineage marker OLIG2, for OPC-specific surface receptor (e.g., platelet-derived growth factor receptor-α (PDGFRα)) and for differentiation markers (e.g., CC1 or MBP). At postnatal day 14, mutants were characterized by dramatic hypomyelination (Fig. 2a, c), reduction of CC1^+^/OLIG2^+^ OLs (Fig. 2b, d), and precocious mortality (Fig. 2e). Ablation of the enzyme at later stages of development, using the *Cnp-Cre* driver line to target OLs, did not significantly reduce myelin content or affect cell number (Supplementary Fig. 3).

**Fig. 3:**
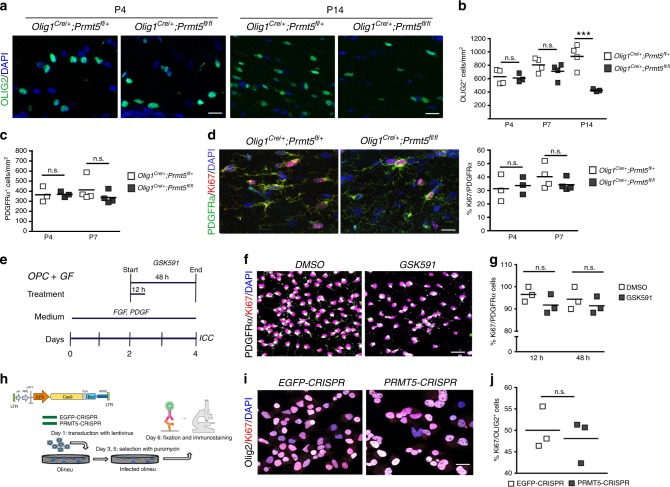
Reduced OPC number in the absence of *Prmt5 *is independent of proliferation. **a** Representative confocal images of P4 and P14 corpus callosum from controls (*Olig1*^*Cre/+*^;*Prmt5*^*fl/+*^) and *Prmt5* mutants (*Olig1*^*Cre/+*^;*Prmt5*^*fl/fl*^) stained for OLIG2 (green) and DAPI (blue). Scale bar: 20 μm. **b**, **c** Quantification of total (**b**) OLIG2^+^ or (**c**) PDGFRα^+^ cell numbers per unit area in the corpus callosum of three (P4 and P14) or four (P7) mutants and four controls at the indicated time points. One-way ANOVA with Bonferroni’s multiple comparison test, ***p < 0.001. **d** Confocal images of corpus callosum from controls (*Olig1*^*Cre/+*^;*Prmt5*^*fl/+*^) and *Prmt5* mutants (*Olig1*^*Cre/+*^;*Prmt5*^*fl/fl*^) stained for PDGFRα (green) KI67 (red), and DAPI (blue). Scale bar: 20 μm. Scatter plots represent the average cell counts in three mice for each genotype at P4 and four mice at P7. One-way ANOVA with Bonferroni’s multiple comparison. **e** Schematics of the experiment with the PRMT5 pharmacological inhibitor, GSK591. **f** Representative confocal images of DMSO-treated or GSK591-treated OPCs stained for PDGFRα (white), KI67 (red), and DAPI (blue). Scale bar: 20 μm. **g** Quantification of proliferating OPC (KI67^+^ PDGFRα^+^) from three biological replicates (one-way ANOVA). **h** Schematic representation of CRISPR knockdown experiment. **i** Representative confocal images of control (EGFP-CRISPR) and knockdown (PRMT5-CRISPR) OliNeu cells stained with antibodies specific for OLIG2 (white), KI67 (red), and DAPI (blue). Scale bar: 20 μm. **j** Quantification of KI67 immunoreactivity measured in at least 50 OLIG2^+^ cells from three biological replicates. Scatter plots indicate values relative to the average of the EGFP-CRISPR group (Student’s *t* test). n.s. nonsignificant

The decreased number of OLIG2^+^ cells was first detected at postnatal day 14 *Olig1*^*Cre/+*^;*Prmt5*^*fl/fl*^ mice and not observed at earlier developmental time points (Fig. 3a, b) and was not accompanied by a reduction of PDGFRα^+^ OPCs (Fig. 3c). Proliferation of OPCs in vivo was not affected by loss of *Prmt5* (Fig. 3d) and this was in contrast with previous reports on the effect of PRMT5 inhibition in cancer cells^40,41^. To further ascertain that PRMT5 did not affect proliferation, we treated primary cultures of OPCs with GSK591, a selective inhibitor of PRMT5 (which inhibits symmetric dimethylation of arginine containing substrates by the PRMT5/MEP50 complex^42^) (Fig. 3e). Progenitors were identified by PDGFRα immunoreactivity and proliferation was measured by counting the number of cells immunoreactive for both KI67 and PDGFRα (Fig. 3f, g). Similar to the in vivo condition, inhibition of PRMT5 enzymatic activity in primary cultured proliferating OPCs did not reduce proliferation (Fig. 3g). To begin characterizing the PRMT5-dependent mechanism of OL cell number regulation, we adopted the CRISPR/Cas9 lentiviral system, which effectively decreased its protein levels (Supplementary Fig. 4a, b). Cell counts at multiple time points after PRMT5-CRISPR lentiviral construct infection revealed reduced numbers compared to EGFP-CRISPR controls (Supplementary Fig. 4c). Downregulation of PRMT5, using this system (Fig. 3h), also did not significantly impact proliferation of the cells (Fig. 3i, j).

Collectively, these results highlight the role of PRMT5 as a key modulator of OPCs early development and differentiation, but not as a regulator of physiological proliferation.

### Decreased progenitor survival and differentiation without PRMT5

**Fig. 4:**
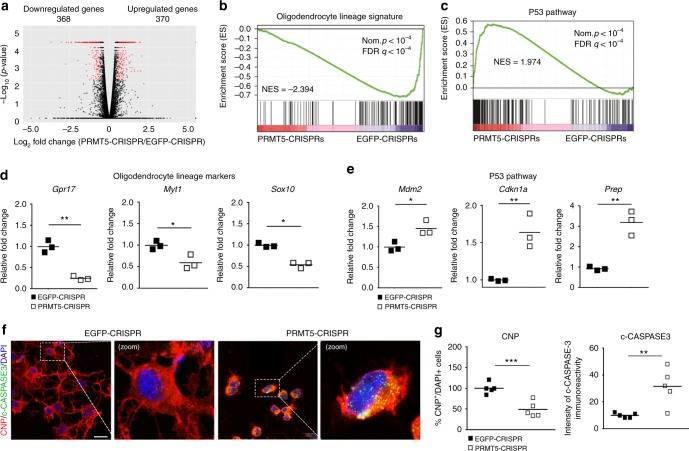
Transcriptional consequences of decreased PRMT5 expression, using CRISPR/Cas9. **a** Volcano plot representing gene expression differences in OPCs between PRMT5-CRISPR knockdown and EGFP-CRISPR controls. Each gene is represented by a dot with the red dots indicating the differentially expressed transcripts that are statistically significant (fold change >1.5, FDR *q* value <0.05). **b**, **c** Gene set enrichment analysis (GSEA) identified **b** oligodendrocyte-specific genes as downregulated and **c** P53 targets genes as up-regulated upon PRMT5 knockdown. **d**, **e** qRT-PCR validation of representative genes in each category, including **d** oligodendrocyte lineage (*Gpr17*, *Myt1*, and *Sox10*) and **e** P53 targets (*Mdm2*, *Cdkn1a*, and *Perp*) genes. Transcripts were normalized to the geo-mean of three housekeeping genes (*18s*, *Wdr33*, and *Pja2*). Scatter plots represent average values of three independent preparations relative to controls. Student’s *t* test, *p < 0.05, **p < 0.01. **f** Representative confocal images of control (EGFP-CRISPR) and knockdown (PRMT5-CRISPR) oligodendrocyte lineage cells cultured in differentiating conditions for 48 h and then stained with antibodies specific for CNP (red), cleaved CASPASE-3 (c-CASPASE3, green), and DAPI (blue). Scale bar: 10 μm. **g** Scatter plots represent average number of CNP^+^ relative to total DAPI^+^ cells and average intensity of cleaved CASPASE-3 staining in five wells from three independent biological replicates in the PRMT5-CRISPR (418 total cells counted) group relative to the EGFP-CRISPR control group (428 total cell counted). Student’s *t* test, **p < 0.01, ***p < 0.001

The transcriptional consequences of PRMT5 depletion in OL lineage cells were further evaluated using RNA-sequencing (RNA-seq) analysis of four biological replicates of PRMT5-CRISPR and EGFP-CRISPR cells, which revealed similar number of reads in the four replicates of each group (Supplementary Table 2). Based on a threshold of fold change >1.5 and false discovery rate (FDR) *q* value <0.05, we identified 368 downregulated and 370 up-regulated genes in the PRMT5-CRISPR cells compared to the control group (Fig. 4a and Supplementary Data 1). When interrogated using the Gene Set Enrichment Analysis (GSEA) software based on curated datasets (for details please see Online Methods), we identified downregulation of “OL signature” genes, and up-regulation of “P53-pathway” genes (Fig. 4b, c).

Among the OL-specific genes, we identified and validated transcripts whose levels were decreased at least 50% in the mutants compared to controls and included the G-protein-coupled receptor *Gpr17* and the transcription factors *Sox10* and *Myt1* (Fig. 4d, Supplementary Data 1, and Supplementary Table 3).

Among the P53 target genes, we identified and validated transcripts whose levels were increased at least 50% in the mutants compared to controls. They included the E3 ubiquitin ligase and negative regulator of P53 *Mdm2*^43^, the growth arrest gene *Cdkn1a* and the pro-apoptotic gene *Perp* (Fig. 4e). Of note, MDM2 is a negative regulator of P53 and yet the protein levels of P53 were up-regulated in cells after PRMT5-CRISPR knockdown (Supplementary Fig. 4a, b). A similar up-regulation of P53 target genes had been previously detected in neural stem cells lacking PRMT5 enzymatic activity and attributed to changes in pre-mRNA splicing of molecules involved in P53 activity (e.g., *Mdm4*)^34^. For this reason, we analyzed differential splicing events using MATS 3.0.6 beta^44^ and identified 215 skipped exon, 35 mutually exclusive exon, 15 alternative 5′ or 12 alternative 3′ splice sites, and 6 retained introns as differentially spliced in the mutants compared to controls (Supplementary Fig. 5c and Supplementary Table 4).

This was distinct from what had been previously reported  for neural stem cells^34^ or OPCs from mutants of other epigenomic regulators^45^. For instance, in contrast to neural stem cells, we did not detect intron-retention events as one of the most affected category and we reasoned that these differences could be explained by transcriptomic differences among distinct cell types. However, despite the fact that *Mdm4* splicing was not significantly altered in *Prmt5*-null OPCs, based on its previously reported role as key regulators of P53 levels in neural stem cells, we asked whether it could also be affected in OPCs. Assessment of *Mdm4* splice variants in OPCs after CRISPR-PRMT5 revealed the presence of a weak band corresponding to the smaller isoform (*Mdm4s*) in the cells lacking *Prmt5* (Supplementary Fig. 5d). Therefore, the effect of *Prmt5* ablation on *Mdm4* splicing in OPCs was modest and did not resemble the strong effect detected in neural stem cells during embryonic development^34^, suggesting that the effect of *Prmt5* loss of function is highly cell type specific.

To further define whether the up-regulation of P53 and its target genes induced apoptosis of OPCs, we stained EGFP-CRISPR and PRMT5-CRISPR primary OPC cultures with antibodies specific for cleaved caspase-3 as a marker of apoptosis, and CNP as a marker of OL differentiation (Fig. 4f). Ablation of *Prmt5* resulted in increased number of apoptotic CNP^+^ cells (Fig. 4g) and impaired differentiation, as even the immunoreactive cells did not display the characteristically branched morphology of mature OLs. Overall, these results suggested that PRMT5 was necessary not only for OPC survival but also for their differentiation.

**Fig. 5:**
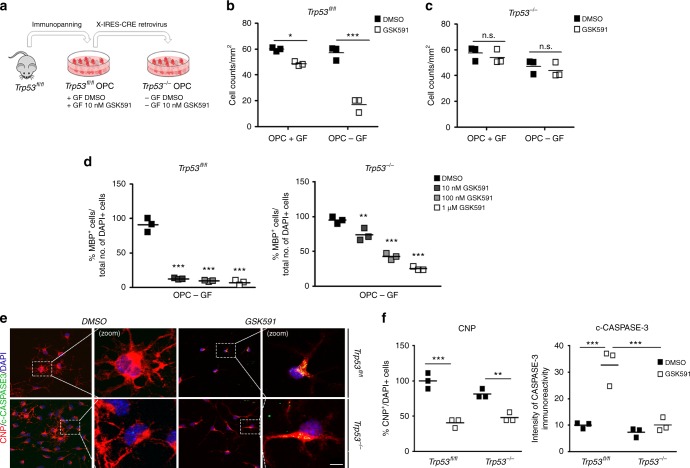
Activation of p53-dependent apoptosis in progenitors treated with PRMT5 inhibitors. Effect of the pharmacological inhibitor of PRMT5 (GSK591) on cell viability and differentiation of primary cultures of OPCs from wild-type (*Trp53*^*fl/fl*^) and p53 mutant (*Trp53*^*−/−*^) mice. **a** Schematics of experimental conditions. **b** Average cell counts from three biological replicates of wild-type (*Trp53*^*fl/fl*^) and (**c**) *Trp53*-null (*Trp53*^*−/−*^) OPC cultures in the presence (+GF) and absence (−GF) of growth factors, and with or without 10 nM GSK591. **d** Average MBP^+^ cell counts from three biological replicates of wild-type (*Trp53*^* fl/fl*^) and p53 mutant (*Trp53*^*−/−*^) OPCs cultured in differentiating conditions in the absence (DMSO) or presence of increasing concentration of GSK591. One-way ANOVA, *p < 0.05, **p < 0.01, and ***p < 0.001. **e** Representative confocal images of wild-type (*Trp53*^*fl/fl*^) and *Trp53-*null (*Trp53*^*−/−*^) OPCs cultured in differentiating conditions, with or without 10 nM GSK591 and stained with antibodies specific for CNP (red), cleaved CASPASE-3 (c-CASPASE3, green), and DAPI (blue) (scale bar = 10 μm). **f** Scatter plots of the average number of CNP^+^ cell relative to total DAPI^+^ cells and average intensity of cleaved CASPASE-3 staining from three biological replicates of GSK591-treated wild-type (228 total cells counted) and *Trp53*-null OPCs (232 total cell counted) relative to untreated wild-type (240 total cells counted) and  *Trp53*-null (256 total cell counted). One-way ANOVA, **p < 0.01, ***p < 0.001. n.s. nonsignificant

To further characterize the mechanism underlying the P53-dependent OPC survival after inhibition of PRMT5 activity, we used ablation of its gene, *Trp53,* in primary OPCs. We obtained OPCs from *Trp53*^*fl/fl*^ mice, infected them with *Cre* retroviruses (recombination was 72%), and used uninfected *Trp53*^*fl/fl*^ cells as controls (Fig. 5a). Because we identified PRMT5 as important for the transition of OPCs between growth arrest and differentiation, we analyzed the effect of the PRMT5 pharmacological inhibitor GSK591 in the presence (to maintain proliferation) or absence of growth factors (to induce growth arrest). Consistent with the phenotype detected in *Prmt5* mutant mice, GSK591 treatment had a minor impact on proliferating OPCs, while it profoundly decreased the number of differentiating cells (Fig. 5b, c). Interestingly, deletion of *Trp53* completely rescued the effect of the PRMT5 inhibitor on survival (Fig. 5b). However, it rescued only partially the number of differentiating OPCs (Fig. 5c). To define whether pharmacological inhibition of PRMT5 induced apoptosis, we stained differentiated cells with antibodies specific for CNP and for cleaved caspase-3 (Fig. 5d). Also, in this case, we detected a profound reduction of the number of cleaved caspase-3-positive CNP^+^ cells in differentiating OPCs lacking *Trp53,* in response to GSK591 treatment. Importantly, the overall number of CNP^+^ cells was not entirely rescued by the absence of P53 (Fig. 5e), thereby suggesting that the effect of PRMT5 inhibitors on differentiation cannot be entirely explained in terms of survival.

Together, these results support the concept that PRMT5 is critical for OPC differentiation and that in its absence cells cannot properly differentiate and therefore are eliminated via a P53-dependent mechanism of apoptosis.

### Histone arginine methylation and lysine acetylation cross-talk

**Fig. 6:**
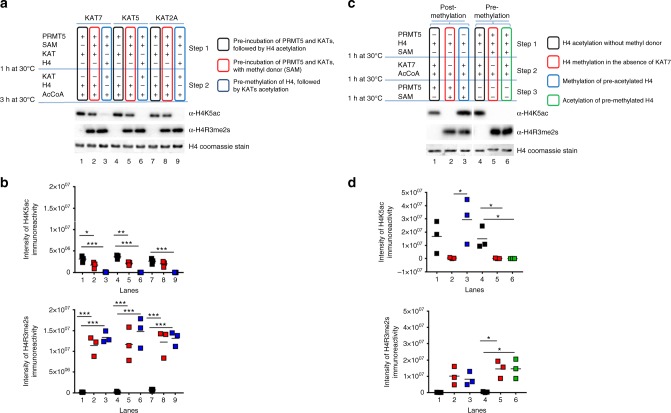
Cross-talk between PRMT5-dependent histone arginine methylation and lysine acetylation. **a** Representative experiment of sequential PRMT5-mediated methylation of arginine residues on histone H4 followed by KAT-dependent acetylation of histone H4 by KAT7 (lanes 1–3), KAT5 (lanes 4–6), and KAT2A (lanes 7–9) in the absence (lanes 1, 4, and 7) or presence (lanes 2, 3, 5, 6, 8, and 9) of methyl donor. The results support the cross-talk between histone H4 arginine methylation and lysine acetylation. The enzymatic activity was evaluated by western blot analysis using antibodies specific for H4K5ac and for H4R3me2s. **b** Scatter plot of the quantification of the immunoreactive bands from three independent western blots for each of the experimental conditions described in **a**. One-way ANOVA with Bonferroni multiple comparison test, *p < 0.05, **p < 0.01, and ***p < 0.001. **c** Representative experiment showing the order of the sequential reactions. Acetylation–methylation reaction (lane 1-3): KAT7-mediated acetylation of H4 followed by symmetric postmethylation by PRMT5 in the absence (lane 1) or presence (lanes 2 and 3) of methyl donor. Ordered methylation–acetylation reaction (lanes 4–6): PRMT5-dependent symmetric premethylation of histone H4 followed by KAT7-mediated acetylation (lanes 4–6). The enzymatic activity was evaluated by western blot analysis using antibodies specific for H4K5ac and for H4R3me2s. **d** Scatter plot of the quantification of the immunoreactive bands from three independent western blots for each of the experimental conditions described in **c**. One-way ANOVA with Bonferroni multiple comparison test, *p < 0.05)

To address the potential mechanisms underlying the defective differentiation of OPCs induced by loss of PRMT5 activity, we then conducted an exploratory proteomic analysis which revealed protein categories related to mRNA processing, transport, and splicing as one of the most representative interacting partners for PRMT5. Interestingly, we also detected the categories related to acetyltransferases and regulation of histone lysine acetylation. Since we had previously shown that decreased bulk of histone acetylation is crucial for the early stages of OPC differentiation into OLs^2,5–9^, we hypothesized the existence of a relationship between PRMT5-dependent methylation of histone arginine and acetylation of lysine residues on histone H4. To explore this relationship, we conducted a series of in vitro reconstitution experiments using methyl and acetyl donors and purified proteins, including PRMT5/MEP50 protein complex, the histone substrate H4, and the histone acetyltransferases KAT7, KAT5, and KAT2a (purchased from SignalChem). Those acetyltransferases were selected because they have the ability to acetylate lysines on histone H4 and they are also expressed in the OL lineage (Supplementary Fig. 6). To understand whether PRMT5-dependent premethylation of the KATs or of the histone H4 interfered with the ability of the histone acetyltransferases to acetylate lysine residues on histone H4, we conducted the first experiment in two steps: premethylation of KATs and H4 followed by the addition of acetyl donor groups and additional histones. Acetylation activity was then assessed by western blot analysis using residue-specific antibodies (Fig. 6a) and the results of three independent experiments were quantified (Fig. 6b).

Acetylation of lysine residue K5 in histone H4 by the acetyltransferases KAT7, KAT5, and KAT2a was reduced only if the histone H4 had been previously methylated on the arginine residue R3 by pre-incubation with PRMT5 (lanes 3, 6, and 9 of Fig. 6a, b). As expected, the pre-incubation of the individual KATs with PRMT5 in the absence of methyl donors did not affect the levels of histone acetylation (as in lanes 1, 4, and 7 of Fig. 6a, b), although a modest reduction of acetylation could be detected if PRMT5 was pre-incubated with KAT5 or KAT7 in the presence of a methyl donor (lanes 2 and 4 in Fig. 6a, b).

Together, these results support the concept that histone lysine acetylation was inhibited by the deposition of H4R3me2s by PRMT5, and not consequent to competition between PRMT5 and the KATs for H4. The mild decrease of histone acetylation detected for KAT5 and KAT7, but not KAT2, also suggests the existence of an additional cross-talk between specific KATs and PRMT5.

To further address whether the inhibitory relationship between arginine methylation and lysine acetylation on histone H4 was reciprocal, we conducted an in vitro sequential enzymatic assay. An ordered acetylation–methylation reaction was obtained by incubating H4, KAT, and Acetyl-coA, to allow lysine acetylation, followed by the addition of PRMT5 and SAM to induce arginine methylation (lanes 1, 2, and 3 in Fig. 6c). Conversely, an ordered methylation–acetylation reaction was obtained by incubating PRMT5 with the methyl-donor SAM and the substrate histone H4 to allow methylation, followed by the addition of KAT and acetyl-coA to induce acetylation (lanes 4, 5, and 6 in Fig. 6c). This experiment was repeated three times and the results quantified (Fig. 6d). While the deposition of acetyl marks on lysine residues by KATs did not prevent symmetric methylation of arginine residues (lane 3 in Fig. 6c, d), the deposition of methyl marks on arginine residues by PRMT5 impaired the ability of acetyltransferases to recognize and acetylate lysine residues (lane 6 in Fig. 6c, d).

Together, these data suggested that PRMT5 has the capacity to decrease the overall output of KATs and predicted that *Prmt5* loss of function would drive a decrease of H4R3me2s marks followed by an increase of histone acetylation in OPCs. Consistent with this hypothesis, decreased levels of H4R3me2s (Supplementary Fig. 7a) and increased levels of H4K5Ac (Supplementary Fig. 7a–c) were detected in OL lineage cells after PRMT5-CRISPR knockdown compared to EGFP-CRISPR controls and also in cells treated with the pharmacological inhibitor GSK591 (Supplementary Fig. 7d, e).

**Fig. 7:**
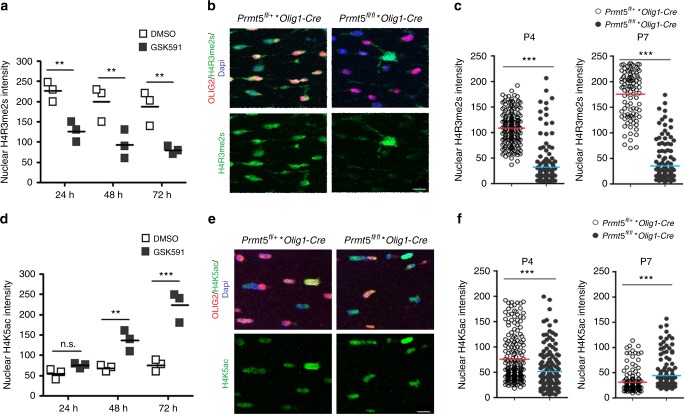
Reduced symmetric histone arginine methylation is followed by increased histone lysine acetylation. **a**, **d** Scatter plot of the pixel intensity of the nuclear immunoreactivity for H4R3me2s (**a**) and H4K5ac (**d**) quantified in an average of 395 cells per experimental condition from three biological replicates of primary OPCs either untreated (DMSO) or treated with the PRMT5 inhibitor (GSK591) for the indicated time points. One-way ANOVA, **p < 0.01, ***p < 0.001. **b** Representative confocal images of P4 and P7 mouse brain sections stained for H4R3me2s (green), OLIG2 (red), and DAPI (blue). Scale bar: 20 μm. **c** Scatter plot of the pixel intensity of nuclear H4R3me2s in OLIG2^+^ cells in control (*Olig1*^*Cre/+*^;*Prmt5*^*fl/+*^) and *Olig1*^*Cre/+*^;*Prmt5*^*fl/fl*^ mutants. A total of 200 cells per genotype per time point was quantified (50 cells/mouse and four mice per condition), with the exception of P4 mutants, where 150 cells were quantified in three mice. Student’s *t* test, ***p < 0.001. **e** Representative confocal images of P4 and P7 mouse brain stained for H4K5Ac (green), OLIG2 (red), and DAPI (blue) (scale bar: 10 μm). **f** Scatter plot of the pixel intensity of nuclear H4K5Ac intensity measured in 200 OLIG2^+^ cells from four control (*Olig1*^*Cre/+*^;*Prmt5*^*fl/+*^) mice at P4 and P7 and from three *Olig1*^*Cre/+*^;*Prmt5*^*fl/fl*^ mutants at P4 and four mutants at P7. Student’s *t* test, ***p < 0.001. n.s. nonsignificant

However, these data did not allow to determine whether the events occurred at the same time or in a sequential fashion. To further explore the temporal relationship between the H4R3me2s and H4K5Ac marks, we performed a time course ex vivo, in primary cultured OPCs treated with the inhibitor and in vivo in brain sections from *Prmt5* mutant mice (Fig. 7). Primary OPCs cultured in chemically defined medium in the absence of growth factor exit from the cell cycle and differentiate. Using this paradigm, we treated OPCs with low concentrations of pharmacological inhibitor GSK591. This induced a significant decrease of H4R3me2s, which occurred within 24 h of GSK591 treatment and persisted over time (Fig. 7a). To further define the effect of *Prmt5* loss of function on histone acetylation and OPC differentiation, we performed the same experiment and showed that increased H4K5 acetylation could only be detected after 48 h of inhibitor treatment (Fig. 7d). Analysis of *Prmt5* mutant mice showed a similar kinetics, with reduced histone arginine methylation detected as early as postnatal day 4 (Fig. 7b, c), followed by increased acetylation, observed at postnatal day 7 (Fig. 7e, f). This is also consistent with the observation that H4R3me2s is mostly cytosolic at postnatal day 4 and nuclear at postnatal day 7 (Fig. 1j).

Thus, the in vivo data in mutant mice, the ex vivo data in primary cultures OPCs, and the in vitro data using purified proteins all consistently showed that the deposition of the R3me2s interferes with acetylation of neighboring lysine residues.

### Rescue of phenotype by histone acetyltransferase inhibitors

**Fig. 8:**
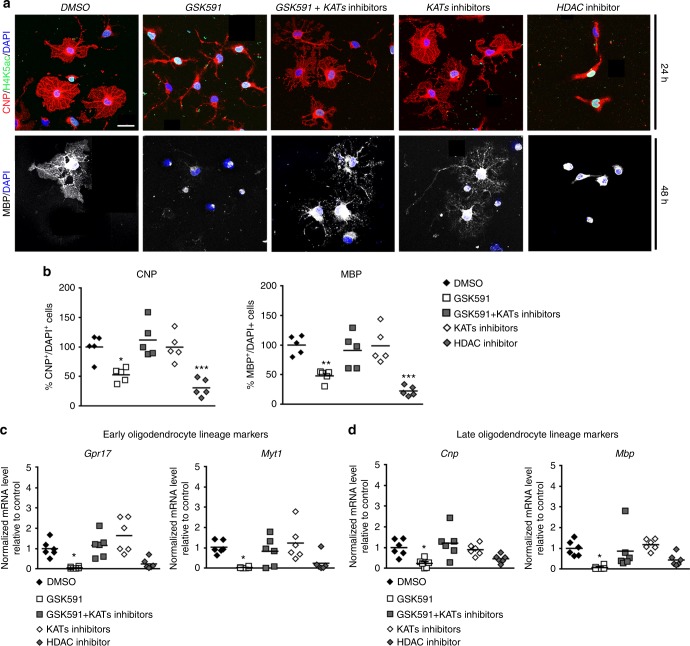
Inhibitors of histone acetyltransferases rescue the effect of PRMT5 loss of activity. **a** Representative confocal images of OPCs cultured in differentiating conditions for 24 and 48 h with DMSO, GSK591, GSK591 + KAT inhibitors, KAT inhibitors (Butyrolactone-3, 100 μM and NU-9056, 0.2 μM) alone, and with the HDAC inhibitor Trichostatin A (TSA, 20 nM). Cells were then stained for CNP (red), MBP (white), H4K5ac (green), and DAPI (blue) (scale bar: 10 μm). **b** Scatter plots of the average number of CNP^+^ (24 h, upper panel) and MBP^+^ (48 h, lower panel) cell relative to total DAPI^+^ cells counted in five wells from three biological replicates. An average number of 367 cells was counted per condition and referred as percentage of the values in the DMSO treated cells. One-way ANOVA with multiple comparison test, *p < 0.05, **p < 0.01, and ***p < 0.001. **c**, **d** qRT-PCR of (**c**) early (*Gpr17*, *Myt1*) and (**d**) late (*Cnp*, *Mbp*) oligodendrocyte lineage gene transcripts measured in six independent preparations and normalized to the geo-mean of three housekeeping genes (*18s*, *Wdr33*, and *Pja2*) and to the internal control (DMSO) at 48 h after treatment. Values were referred to those of the internal control samples (DMSO). One-way ANOVA with Bonferroni multiple comparison test performed for each gene relative to control (*p < 0.05, **p < 0.01, and ***p < 0.001). *n* = 6 independent preparations

The inability of OPCs to differentiate in the absence of PRMT5 enzymatic function and the inverse relationship between histone arginine methylation and acetylation was reminiscent of previous data on impaired OPC differentiation caused by treatment with HDAC inhibitors^5–8^ and led to the hypothesis that KAT inhibitors could rescue the phenotype caused by *Prmt5* loss of function. To test this hypothesis, we performed an ex vivo rescue experiment and asked whether inhibitors of KAT activity (i.e., Butyrolactone-3^46^ and NU-9056^47^) rescued the phenotype induced by lack of PRMT5 activity. OPCs were treated with the PRMT5 inhibitor GSK591, in the absence or presence of the KAT inhibitors. In addition, we included the histone deacetylase inhibitor Trichostatin A (TSA), to further determine whether the effect detected with GSK591 was similar to that previously reported^5–9^. Differentiation was assessed by staining differentiating OPCs with antibodies for CNP or MBP and assessing immunoreactivity and morphological complexity, while histone acetylation on lysine residues was determined by staining with antibodies specific for acetylated H4K5 (Fig. 8a, b). Treatment with TSA mimicked the effect of GSK591, thereby supporting the concept that the PRMT5 effect on OL differentiation is the likely consequence of increased histone acetylation. Consistently, addition of the KAT inhibitors to OPCs treated with the PRMT5 inhibitor prevented the increase of H4K5 acetylation and rescued the differentiation phenotype, as shown by their morphology (Fig. 8a), the number of cells immunoreactive for OL differentiation markers (Fig. 8b), and their pattern of gene expression (Fig. 8c, d). By rescuing differentiation, the KAT activity inhibitors had also a significant protective effect that was detected as decreased expression of P53-responsive genes (Supplementary Fig. 9).

Together, these data highlight the importance of the PRMT5-mediated cross-talk between histone methylation and acetylation and the role it plays in OL differentiation. In conclusion, this study reveals a biologically significant role for PRMT5 in OL biology, by unveiling the existence of an important cross-talk between symmetric arginine methylation and lysine acetylation on histone tails regulating differentiation and survival of the OPCs.

## Discussion

OLs, the myelin-forming cells of the CNS, differentiate from progenitor cells, and the epigenetic and transcriptional changes defining this transition have been the subject of extensive investigation^48^. Despite general agreement on the role of histone deacetylation for the early stages of differentiation^2,5–7,9,49^, the events preceding this important hallmark of differentiation have not been thoroughly characterized. Here we focus on PRMT5, an enzyme which is expressed at high levels in OL lineage cells^17,18^ and in gliomas^28^ and characterize the functional significance of a relatively under-investigated modification of nucleosomal histones^50^.

Symmetric methylation of histone arginine residues catalyzed by PRMT5 emerges as an important negative regulator of histone acetylation, during the differentiation of OPCs. We and others have previously shown that proliferating OPCs are characterized by high levels of nuclear histone acetylation. This likely reflects the absence of PRMT5 from the nucleus of these cells and  its localization -  together with newly formed histone proteins - in their cytoplasm ^51–54^. This cytosolic localization is consistent with previous reports in ES cells^55^. As OPCs stop proliferating and exit from the cell cycle, transcriptional regulators of this transition are also subject to nucleo-cytoplasmic shuttling: with E2F1 becoming mostly cytosolic^56^ and P53 becoming mostly nuclear^57^. This is when PRMT5 becomes nuclear and symmetric histone arginine methylation is detected in the nucleus of differentiating OPCs. It has been previously suggested that PRMT5-dependent methylation of specific arginine residues^58^ on P53 impacts its subcellular localization and promotes cell cycle exit. Our data do not support this model in OPCs, as nuclear P53 and high levels of P53 target gene expression were still detected in OPCs lacking PRMT5 enzymatic activity. In addition, we did not detect any prominent effect of PRMT5 on proliferating OPCs, and this is in contrast with previous reports of growth arrest consequent to PRMT5 inhibition in tumor cells^40,41^. The discrepancy between our results obtained in OPC studied in physiological conditions and previous reports in cancer cells suggest that the previously reported direct interaction between PRMT5 and P53 may not be physiologically relevant for OPCs.

The detection of P53-dependent apoptosis after pharmacological inhibition or CRISPR/Cas9 or genetic ablation in mice was only detected in OPCs kept in differentiating conditions and resulted in the elimination of those cells that had started to differentiate into CNP^+^ cells. The effect was only minimally detected in proliferating cells and suggested that the decreased survival of OLs in the absence of PRMT5 activity was consequent to the elimination of an aberrantly differentiating cell.

The inverse relationship between symmetric arginine methylation and histone acetylation reported in this study is of particular importance, given the well-documented role of histone deacetylation in OPC differentiation^2,5–9^. We report here that PRMT5-mediated deposition of R3me2s mark on histone H4 precludes the acetylation of lysine K5 acetylation, thereby suggesting a mechanism of epigenetic repression due to decreased histone acetylation. Our model suggests that PRMT5 activity blocks re-acetylation of the histones once OPC differentiation starts, and therefore shifts the dynamic equilibrium of histone acetylation towards the deacetylated state. This is of great relevance, especially when considering the fact that H4K5Ac has been mostly related to transcriptional activation and shown to interfere with chromatin compaction in yeasts^59^. A dramatic reduction of H4R3me2s was detected in the nucleus of differentiating OPCs treated with pharmacological inhibitors of PRMT5, or after CRISPR knockdown and in the brain of *Prmt5* conditional knockout mice. Reduced or absent PRMT5 activity was followed by increased nuclear histone acetylation and therefore suggested that KATs could continue to acetylate histones even after differentiation had ensued. In vitro studies with purified enzymes and histone proteins further indicated that PRMT5 activity interfered with the enzymatic activity of the KAT proteins, possibly by impacting the recognition of lysine residues, as symmetric methylation of arginine R3 on histone H4 prevented further acetylation of lysine K5, while the reverse was not true.

 In conclusion, this manuscript identifies a unique function of PRMT5 in differentiating OPCs: orchestrating the cross-talk between histone symmetric arginine methylation and prevent re-acetylation of critical lysine residues in cells which require histone deacetylation and chromatin compaction to achieve a fully differentiated state. This interpretation was further supported by the similar effect of HDAC inhibitors and PRMT5 inhibitors on OPC differentiation and by the rescue of differentiation using KAT inhibitors. Impaired cross-talk in cells lacking PRMT5 induced a heterochronic acetylation of lysine residues in cells starting a differentiation program and this led to elimination by a P53-dependent mechanism of apoptosis. This function is selective for OPC in physiological conditions, as highlighted by the severe effects on developmental myelination detected in mice with OL lineage-specific ablation of *Prmt5*.

Consistent with the possibility that the PRMT5-dependent effect on OPC differentiation was consequent to its enzymatic activity on histone arginine residues, which in turn prevented re-acetylation during differentiation, we showed that pharmacological inhibitors of KAT histone acetyltransferases were sufficient to overcome the differentiation block and rescue both survival and differentiation.

Together, these data suggest a critical role for PRMT5 in modulating the levels of histone acetylation in OPCs at the critical temporal window of exit from the cell cycle and onset of differentiation. This also explains why later ablation of PRMT5 (once the cells have started to differentiate) does not result in a hypomyelinating phenotype and may explain why a decline of PRMT5 levels at later stages of development is compatible with physiological function.

## Methods

### Primary OPC cultures

Primary mouse OPCs were isolated from the brain of C57BL/6 mice at postnatal day 7 through immunopanning with a rat anti-mouse CD140a antibody, recognizing PDGFRα, as previously described^2^ and were cultured in SATO medium (Dulbecco's modified Eagle's medium (DMEM), 10 mg/ml bovine serum albumin (BSA), 10 mg/ml apotransferrin, 1.6 mg/ml putrescine, 6 ng/ml progesterone, 4 μg/ml selenium, 5 mg/ml insulin, 1 mM sodium pyruvate, 2 mM l-glutamine, 100 U/ml penicillin, 100 g/ml streptomycin, B27 Supplement, 5 mg/ml *N*-acetyl-cysteine, Trace Element B, 10 μg/ml biotin, 50 mM forskolin) supplemented with PDGF-AA (10 ng/ml) and basic fibroblast growth factor (bFGF) (20 ng/ml). The growth factors were removed and T3 (60 nM) was added to induce differentiation. *Trp53*^*fl/fl*^ OPCs were cultured as described above and then infected with an X-IRES-CRE retrovirus to obtain *Trp53*^*−/−*^ OPCs.

OPCs were treated with or without the PRMT5 inhibitor (GSK591, 10 nM, 100 nM, 1 μM). For the rescue experiment, the KAT inhibitors (Butyrolactone-3, 100 μM and NU-9056, 0.2 μM) were added after 30 min of incubation with 10 nM GSK591. Control cultures were treated with dimethyl sulfoxide (DMSO). GSK591 was obtained from the Structural Genomics Consortium (SGC), while Butyrolactone-3 and NU-9056 were purchased from Cayman Chemicals and Tocris Bioscience, respectively.

### OliNeu cell line

The OPC cell line OliNeu (a gift by Jackie Trotter)^60^ was cultured in the ODM medium (DMEM, 100 g/ml BSA, 100 g/ml apotransferrin, 16 g/ml putrescine, 0.06 ng/ml progesterone, 40 ng/ml sodium selenite, 5 g/ml insulin, 1 mM sodium pyruvate, 2 mM l-glutamine, 100 U/ml penicillin, 100 g/ml streptomycin) supplemented with 1% horse serum on plates coated with poly-d-lysine.

### Primary tumor cell lines

Primary tumor cell lines (the JF line for proneural glioma cells expressing wild-type *Trp53* and the dfYB line for *Trp53*-null proneural glioma cells) and T*p53*^fl/fl^ mice were provided by Dr. Peter Canoll’s laboratory at Columbia Presbyterian Medical Center. The tumor cell lines were derived as described previously^25,26,61^. Briefly, a retrovirus encoding the cDNA for an OPC mitogen (PDGFB) and the cDNA for the CRE recombinase was injected into the subcortical white matter of adult transgenic mice harboring floxed tumor suppressor genes (i.e., *Pten* in the JF line and *Pten* and *Trp53* in the dfYB line). Overexpression of the PDGF-BB mitogen, combined with the deletion of the tumor suppressor gene(s) was reported to be sufficient to the formation of brain tumors with the histopathologic and molecular features of proneural glioblastomas^25,26^. Cells derived from tumor tissues were cultured in medium containing DMEM (1 mM sodium pyruvate, 2 mM l-glutamine), FBS, N2 Supplement, PDGF-AA (10 ng/ml), bFGF (20 ng/ml), and antibiotic–antimycotic solution.

### Generation of the lenti-CRISPR/Cas9 knockdown system in 293T cells

The lenti-CRISPR-v2 vector was obtained from Addgene, which was a gift from Feng Zhang (Addgene plasmid # 52961). The single guide RNA (sgRNA) targets were obtained via the online program generated by Feng Zhang’s laboratory (http://crispr.mit.edu/). The sgRNA target sequence of PRMT5-CRISPR-2 (GAATTGCGTCCCCGAAATAG) falls on the exon 1 of the mouse *Prmt5* gene, while that of PRMT5-CRISPR-3 (CCCGCGTTTCAAGAGGGAGT) falls on the exon 2. Two sgRNAs targeting the DNA sequence of the *EGFP* gene (GGGCGAGGAGCTGTTCACCG for EGFP-CRISPR-1 and GAGCTGGACGGCGACGTAAA for EGFP-CRISPR-2) were also cloned into the same vector, respectively, as used as control. The cloning was performed according to the Addgene guideline and the original paper^62^. The 293T cells were cultured in the 293T medium (DMEM, 1 mM sodium pyruvate, 2 mM l-glutamine) supplemented with 10% FBS. The lenti-CRISPR viruses were produced by transfecting the lenti-CRISPR/Cas9 plasmids along with two packing plasmids (psPAX2 and pMD2.G which were acquired from Addgene). For each 10-cm dish of 293T cells, 10 μg of the lenti-CRISPR/Cas9 plasmids, 6 μg of the psPAX2 plasmid, and 2 μg of the pMD2.G plasmids were transfected into the 293T cells using polyethylenimine. Tissue culture media were refreshed 15 h after transfection and media containing viruses were harvested 45 h after transfection. Viruses were concentrated using the Lenti-X™ concentrator kit (Clontech).

### *Prmt5* knockdown in cells

One million primary mouse OPCs or 0.5 million of OliNeu cells were split into each 10-cm dish a day before virus infection. Virus infection was performed by adding the concentrated virus into the tissue culture medium of the cells to be infected, supplemented with polybrene (2 μg/ml for primary OPCs or 4 μg/ml for OliNeu). Virus-containing media were replaced by fresh ones 8 h after infection. Puromycin (0.2 μg/ml for primary OPC or 1 μg/ml for OliNeu and glioma cells) was added to the medium 2 days after infection to select the infected cells. Infected cells were harvested 6 days after infection for experimental analysis. The experiment was independently replicated by two investigators in the lab.

### *Prmt5* conditional knockout in mice

All animal experiments were approved by and performed according to the guidelines set forth by the US Public Health Service in their policy on Human Care and Use of Laboratory Animals. Mice were maintained in a pathogen-free environment at the animal facility of Mount Sinai Medical Center and of the Advanced Science Research Center of College University of New York (CUNY). All procedures received prior approval from the Institutional Animal Care and Use Committee of each institution. The C57BL/6^−^ *Prmt5*^−^ *flox* mice^34^ were obtained from Dr. Ernesto Guccione (Institute of Molecular and Cell Biology, A*STAR Singapore). *Olig1-cre* line (stock number 011105) was obtained from the Jackson Laboratory and backcrossed to the C57BL/6 background. Experimental animals were generated by crossing the homozygous floxed *Prmt5* line (*Prmt5*^fl/fl^) with the heterozygous *Olig1*-*cre* line. F1 offspring from the crossbreed that were heterozygously floxed and Cre positive (*Olig1*^*cre/+*^;*Prmt5*^*fl/+*^) were intercrossed with other F1 offspring that were heterozygously floxed and Cre negative (*Olig1*^*+/+*^; *Prmt5*^*fl/+*^) to achieve the following experimental genotypes: conditional knockout (*Olig1*^*Cre/+*^;*Prmt5*^*fl/fl*^), floxed control (*Olig1*^*+/+*^;*Prmt5*^*fl/fl*^), and Cre control (*Olig1*^*cre/+*^;*Prmt5*^*fl/+*^). *Prmt5* conditional knockout mice, heterozygotes, and wild-type littermates were checked daily for growth, clinical signs, and survival.

### Subcellular protein fractionation

Subcellular fractionation of cellular proteins consisted of: lysis in hypotonic buffer (10 mM HEPES, pH 7.9, 1.5 mM MgCl_2_, 10 mM KCl) supplemented with 0.5 mM dithiothreitol (DTT), 1 mM phenylmethylsulfonyl fluoride (PMSF), and protease inhibitor cocktail freshly prepared (at 4 °C for 15 min), followed by 0.5% NP40 treatment (for 10 s) to disrupt the cell membranes. Lysates were then centrifuged at 1500 × *g* for 10 min at 4 °C to separate the cytoplasmic components (supernatant) from the nuclei-enriched fractions (pellets). Volumes (0.11) of 10× cytoplasmic extraction buffer (0.3 M HEPES, 1.4 M KCl, and 30 mM MgCl_2_) was added to the supernatant and sonicated for 30 s ON/OFF for 5 min at high power in Bioruptor (Diagenode). After centrifugation at 16,000 × *g* for 10 min at 4 °C, the soluble fraction was collected as a cytoplasmic extract. The pellet obtained after the first centrifugation was then washed twice with hypotonic buffer with 0.5% NP40 (the wash out was also saved for control in western blot). Washed pellets were further extracted by re-suspension in a hypertonic buffer (20 mM HEPES, pH 7.9, 1.5 mM MgCl_2_, 420 mM NaCl, 25% (v/v) glycerol, and 0.2 mM EDTA) supplemented with 0.5 mM DTT, 1 mM PMSF, 10 μM TSA, phosphatase inhibitor cocktail, protease inhibitor cocktail, and benzonase (Sigma), sonicated and supernatant collected as a nuclear fraction.

### Histone extraction and purification

Histones were extracted by using the acid extraction method^63^. Cell pellets were incubated in a hypotonic lysis buffer containing 10 mm Tris-HCl, pH 8.0, 1 mMKCl, 1.5 mM MgCl_2_, 1 mM DTT, 0.4 mM PMSF, and protease and phosphatase inhibitors for 30 min on rotator at 4 °C. Nuclei were isolated by spinning down the solution for 10 min at 10,000 x *g* and dissolving and incubating pellets for 1.5 h in 0.4 N H_2_SO_4._ After centrifugation at 16,000 x *g* for 10 min, nuclear debris were removed and acid-soluble histones were then precipitated using trichloroacetic acid and re-suspended in water.

### Histone tail peptides arrays and western blot

Characterization of antibodies against histone PTMs used in the study was performed using a Histone Peptide Array (Active Motif, 13005). Briefly, the array was blocked in TBST buffer (10 mM Tris-HCl, pH 8.3, 0.05% Tween-20, 150 mM NaCl) containing 5% non-fat dried milk at 4 °C overnight. The membrane was washed with TBST for 5 min and incubated with a primary antibody. After three TBST washes (10 min each at room temperature), the array was incubated with horseradish peroxidase-conjugated secondary antibody. The membrane was then submerged in ECL developing solution (GE Healthcare, RPN2232) and the data were quantified using the array analyzer software (Active Motif). Western blot was performed after sodium dodecyl sulfate-polyacrylamide gel electrophoresis separation, followed by wet transfer of the proteins into a polyvinylidene difluoride membrane and incubated with primary antibodies. Ponceau S solution (Sigma) was used according to the manufacturer’s instruction. Peroxidase-conjugated secondary antibodies and the ECL Prime Wester Blotting Detection Reagent Kit (GE Healthcare, RPN2232) were then used to develop the membrane. Statistical analysis was performed using one-way analysis of variance (ANOVA) followed by Bonferroni’s post hoc comparisons tests. List of the details related to the antibodies used for histone peptide array and western blot analysis is provided in Supplementary Table 1. The experiment was independently replicated in the lab by two investigators.

### Immunocytochemistry and immunohistochemistry

List of the details related to the antibodies used for immunocytochemistry and immunohistochemistry is provided in Supplementary Table 1. Cells for immunocytochemistry were fixed with 4% paraformaldehyde (PFA) for 20 min at room temperature and then the membrane was permeabilized with 0.1% (vol/vol) Triton X-100 (Fisher Scientific). Incubation with blocking solution (5% normal goat serum) was performed at room temperature for 45 min. Primary antibodies were applied overnight at 4 °C followed by incubation of appropriate secondary antibodies conjugated with fluorophores. Confocal images were captured using the Zeiss LSM-800 system. Quantification of the immunofluorescent intensity was done using ImageJ. At postnatal days 4 and 7 animals were sacrificed and brain tissues were removed and immersion fixed in 4% PFA for 72 h at 4 °C. At postnatal day 14, mice were anesthetized and perfused with 4% PFA. After tissue processing and paraffin embedding, sections of 5–7 μm were cut. To perform immunohistochemistry, sections were de-paraffinized, immersed in 10 mM citrate buffer, pH 6.0, for 10 min in the microwave at 650 W, followed by blocking with 10% normal goat serum, before overnight incubation of primary antibodies at 4 °C. Appropriate secondary antibodies conjugated with fluorophores were used the following day to complete the staining. DAPI (4′,6-diamidino-2-phenylindole) was used as a nuclear counterstain.

Confocal images were captured using the Zeiss LSM-800 system. Quantification of the immunofluorescent intensity was done using ImageJ. Experiments were independently replicated in the lab by two or three investigators.

### RNA extraction, RT-PCR, and RNA-seq

Total mRNAs from cells was extracted by lysing the starting materials with TRIzol^®^ (Thermo, 15596026) followed by RNA extraction using the RNeasy Mini Kit (Qiagen, 74106). For reverse transcription-polymerase chain reaction (RT-PCR), cDNA was synthesized with the qScript cDNA Synthesis Kit (Quantabio, 95047). The RT-PCR for *Mdm4* was performed in Ernesto Guccione’s laboratory as previously reported^34^. Quantitative real-time reverse transcriptase PCR (qRT-PCR) was performed using the PerfeCTa SYBR Green FastMix ROX reagent (Quantabio, 95072) and run at the quantitative PCR core facility at the Icahn School of Medicine at Mount Sinai and at the Epigenetic Core facility of the Advanced Science Research Center of College University of New York (CUNY). For RNA analysis, total RNA was used to prepare libraries using the TruSeq Total RNA Stranded (ribo-gold) Kit (Illumina) and sequenced using HiSeq 2500 (high output) sequencer (Illumina) in the 2 × 125 bp mode. Average number of total reads was 61,602,313.25 for EGFP-CRISPR cells (range: 56,743,181–6,6929,862) and 63,379,315.75 for PRMT5-CRISPR cells (range: 59,009,841–68,576,976). Average and total number of reads for each experiment and single condition are listed in Supplementary Table 2. The sequenced reads were mapped to mm9 version of the mouse genome from the University of California Santa Cruz (UCSC) genome database using TopHat version 2.0.12^64^ with the aligner Bowtie^65^ with their default parameters and by supplying the gene model annotations from Ensemble (version NCBIM37.65). Differential expression analysis was performed by Cuffdiff 2.2.1 with threshold of FDR <0.05. A total of 1890 genes was used to perform the GSEA analysis^66^ using the curated gene datasets C2 and C5. From the initial 1890 genes, we identified the top genes (i.e., 370 up-regulated and 368 downregulated) with a fold change >1.5 (Supplementary Data 1) in either direction (cutoff log 2 FC < 0.5845 < ). A Volcano plot of gene expressions (FPKM) was generated using R program. qRT-PCR data were normalized to the geo-mean of three housekeeping genes: *18s*, *Wdr33*, and *Pja2*. Validation of the results was performed using qRT-PCR using specific primers (Supplementary Table 3). RNA extraction and qRT-PCR was independently replicated in the lab by three investigators. To determine differential splicing events (Supplementary Table 4), MATS 3.0.6 beta^67^ was used for counting junction reads and reads falling into the tested region within ENSEMBL version 65 gene definitions. Four individual preparations for each condition (i.e., four CRISPR-PRMT5 cells and four CRISPR-EGFP control) were analyzed individually, and only significant events occurring in at least two replicates were considered. Splicing events were labeled significant if the sum of the reads supporting a specific event exceeded 10 reads, the *P* value was <0.05, and the minimum inclusion level difference as determined by MATS was >0.2. Total events (the sum of the reads supporting a specific event exceeded 10 reads, the *P* value was <0.05, and the minimum inclusion level difference as determined by MATS was >0.2).

### In vitro biochemistry assays

For the sequential PRMT5 methylation-KAT acetylation assay and the ordered acetylation–postmethylation/premethylation–acetylation assay, 800 ng of PRMT5-MEP50, 50 ng of the specified KAT, 1 μg of histone H4, and 2 μl of 1 mM acetyl-CoA was added to the acetylation buffer (50 mM Tris, pH 8, 10 mM KCl, 2 mM DTT, 5 mM MgCl_2_) to a total volume of 20 μl and incubated at 30 °C as indicated.

PRMT5 and MEP50 were co-expressed in insect cells and the resulting complex isolated by affinity purification and gel filtration^68^. Full-length PRMT5 was expressed as FLAG fusion protein (FLAG-PRMT5). Standard baculovirus expression using a modified version of the Bac-to-Bac system protocol (Life Technologies) was used to generate virus for each clone. Fermentations of PRMT5 and MEP50 (at 1:1 ratio) in Sf9 cells were harvested by centrifugation and pellets were re-suspended in 50 mM Tris, pH 7.5, 300 mM NaCl, 10% (vol/vol) glycerol, 0.1% (wt/vol) Triton X-100, and stirred at 4 °C for 30 min. The homogenate was clarified by centrifugation and the supernatant was gently shaken with 10 ml of anti-FLAG resin (Sigma; A2220) for 3 h, followed by an initial wash with 50 mM Tris, pH 7.5, 500 mM NaCl, 10% (vol/vol) glycerol, 0.1% (wt/vol) Triton X-100, and two subsequent washes without Triton. The PRMT5:MEP50 complex was eluted with 0.1 mg/ml of FLAG peptide (Sigma; F3290) in the same buffer, concentrated, and passed over a size-exclusion chromatography column (16/60 S300; GE LifeSciences) equilibrated in a buffer containing 10 mM HEPES, pH 7.5, 0.15 M NaCl, 10% (vol/vol) glycerol, and 2 mM DTT. Recombinant histone H4 was expressed in bacteria and extracted under denaturing conditions^69^. Briefly, cells were grown in 2 TY medium containing 16 g/l Bacto Tryptone, 10 g/l yeast extract, 5 g/l NaCl, 100 mg/l ampicillin, and 25 mg/l chloramphenicol. Expression was induced at an A600 of 0.8 by the addition of isopropyl-*b*,d-thiogalactopyranoside to a concentration of 0.4 mM and the culture was incubated for another 2.5 h. Bacteria were lysed and the resulting pellet containing the inclusion bodies dissolved under denaturing conditions, by primary incubation in 1 ml of DMSO for 30 min at 22 °C. A 50 ml volume of a 6 M guanidinium hydrochloride solution, containing 20 mM sodium acetate (pH 5.2), 1 mM DTT, was added slowly and unfolding was allowed to proceed for 1 h at 22 °C under gentle mixing. The dissolved inclusion bodies were subjected to gel filtration and the elution of protein monitored by absorbance. The resulting histone was dissolved in water and assayed for purity and concentration via Coomassie staining. KATs were purchased from SignalChem. Western blots were subsequently carried as described before. The list of the antibodies used for the assay is provided in Supplementary Table 1.

### Data availability

All sequencing and microarray data that support the findings of this study have been deposited in the National Center for Biotechnology Information Gene Expression Omnibus (GEO) and are accessible through the GEO Series accession number GSE94067. All other relevant data are available from the corresponding author on request.

## Electronic supplementary material

Supplementary Information

Description of Additional Supplementary Files

Supplementary Data 1
