# Supplementary material for: PRMT5-mediated regulation of developmental myelination
Source: Nat Commun. 2018 Jul 19;9:2840. doi: 10.1038/s41467-018-04863-9 (PMC6053423; doi:10.1038/s41467-018-04863-9)
Supplement: Supplementary file 1 — Supplementary Information [file 41467_2018_4863_MOESM1_ESM.pdf]

# PRMT5-mediated regulation of developmental myelination

Scaglione *et al.*

**<u>Supplementary Figures 1-9</u>**

**<u>Supplementary Tables 1-4</u>**

**<u>Supplementary References</u>**

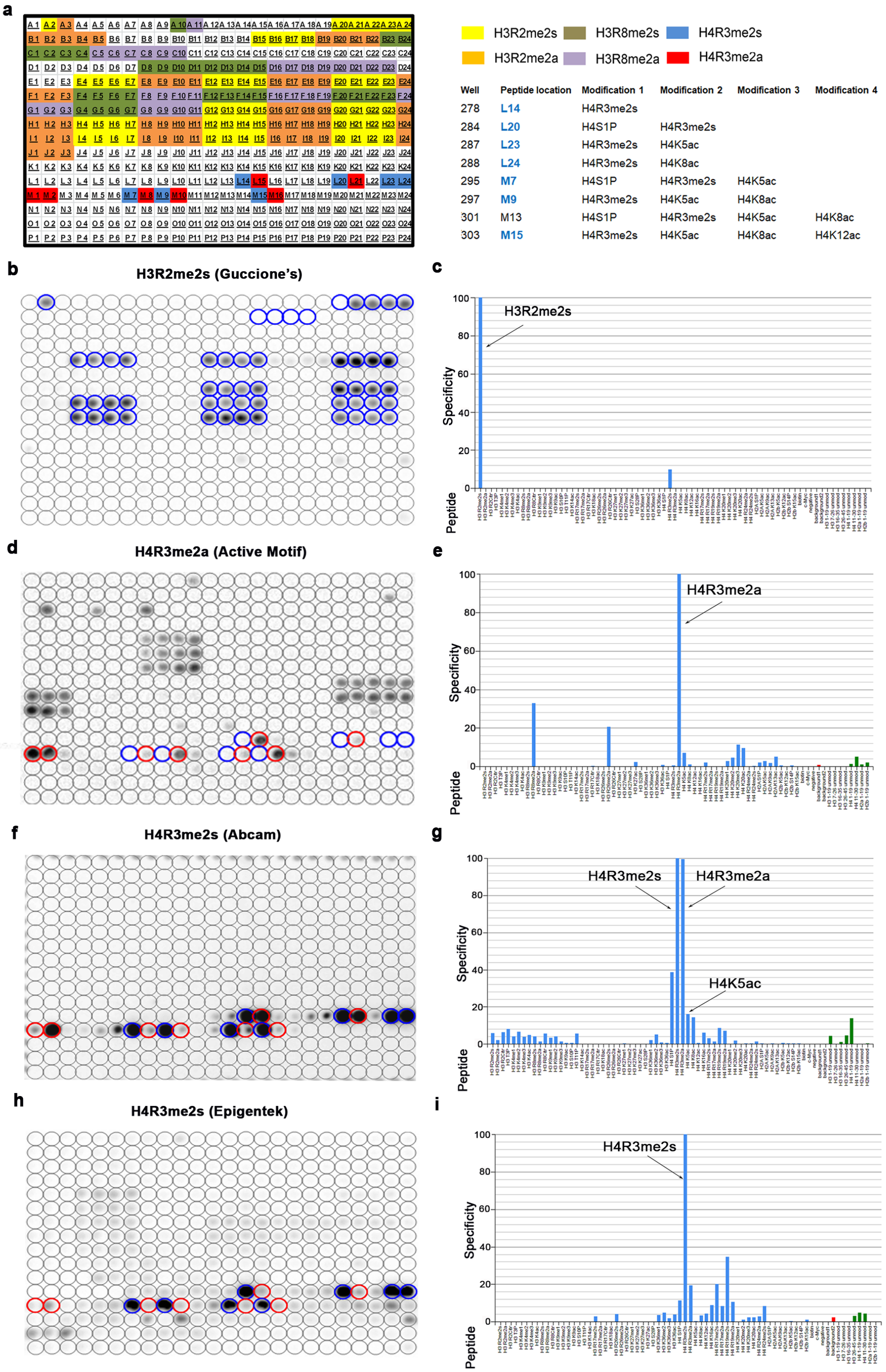


| Well | Peptide location | Modification 1 | Modification 2 | Modification 3 | Modification 4 |
|---|---|---|---|---|---|
| 278 | L14 | H4R3me2s | | | |
| 284 | L20 | H4S1P | H4R3me2s | | |
| 287 | L23 | H4R3me2s | H4K5ac | | |
| 288 | L24 | H4R3me2s | H4K8ac | | |
| 295 | M7 | H4S1P | H4R3me2s | H4K5ac | |
| 297 | M9 | H4R3me2s | H4K5ac | H4K8ac | |
| 301 | M13 | H4S1P | H4R3me2s | H4K5ac | H4K8ac |
| 303 | M15 | H4R3me2s | H4K5ac | H4K8ac | H4K12ac |

**Supplementary Figure 1. Validation of antibody specificity using a modified histone peptide array.** (**a**) Outline of the antibody array from Active Motif. Each well contains a unique combination of histone H3 or H4 peptides with distinctive post translational modifications, as detailed in the Methods section. Histone peptides containing H4 methylated arginine residues or a combination of different post-translational modifications, are highlighted in the table with different colors (yellow for symmetric methylation of R2 in histone H3, red for asymmetric methylation of R3 in histone H4, blue for symmetric methylation of the same residue). The

corresponding position in the array with the relative coordinates and specific examples are indicated. For instance H4R3me2s modification is recognized by the corresponding antibody as the only modification in the histone peptide in the well identified by the coordinates L14. The same antibody, can identify the same post-translational modification also if additional modifications are present, such as acetylation of residue H4K5 in well L23 or acetylation of H4K8, as shown in well L24, or in the presence of multiple acetylated lysine residues, as shown for wells M7, M9, M13 and M15. Multiple antibodies specific for symmetrically or asymmetrically methylated arginine residues in histone H3 or histone H4 were tested using the modified histone peptide array. The results for the antibody specific for H3R2me2s provided by Ernesto Guccione are shown in (**b**). The light purple circles, identify positions in the array containing peptides harboring H3R2me2s.The results for the antibodies specific for H4R3me2a, purchased from Active Motif are shown in (**d**). Those for the antibodies specific for H4R3me2s purchased from Abcam are shown in (**f**) and those for the antibodies purchased from Epigentek are shown in (**h**). In (**d**, **f**, **h**) the red circles identify H4R3me2a and the blue circles identify the H4R3me2s immunoreactive dots. In (**c,e,g,i**) the bar graphs represent the output of the "Multiple Peptide Average" option, calculated using the "Array Analyze Software" provided by Active Motif. The specificity of each antibody is calculated by computing the average intensity of all the spots of the dot blot in positions containing a given mark, divided by the average intensity of all the spots of the array not containing the mark.

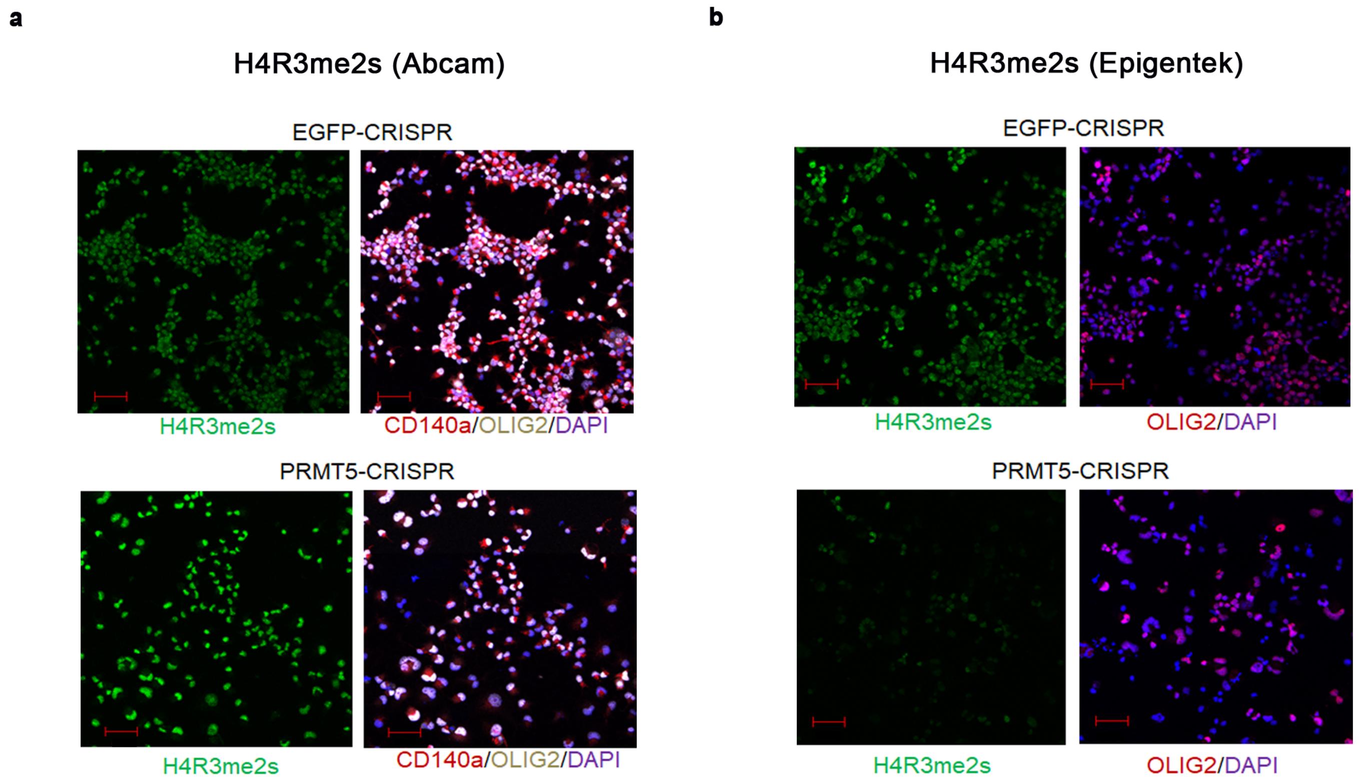


**Supplementary Figure 2. Validation of H4R3me2s antibody specificity for immunocytochemistry using cells with CRISPR/Cas9 targeting of PRMT5.** Representative confocal images of oligodendrocyte progenitor cells with (PRMT5-CRISPR) or without (EGFP-CRISPR) targeting of PRMT5 using lentiviral vectors. Effective knockdown of PRMT5 in OPC was evaluated by immunocytochemistry using antibodies specific for OLIG2 (gray in **a** and red in **b**), for PDGFR$\alpha$ (CD140a antibody in red in **a**) and for H4R3me2s (green) from Abcam (**a**) or Epigentek (**b**). DAPI (blue) as nuclear counterstain. Note that the Abcam antibody identifies immunoreactive cells even after knockdown of PRMT5, which is enzy placing the H4R3me2s mark, while the Epigentek antibody shows greater selectivity. Scale, 50$\mu$m.

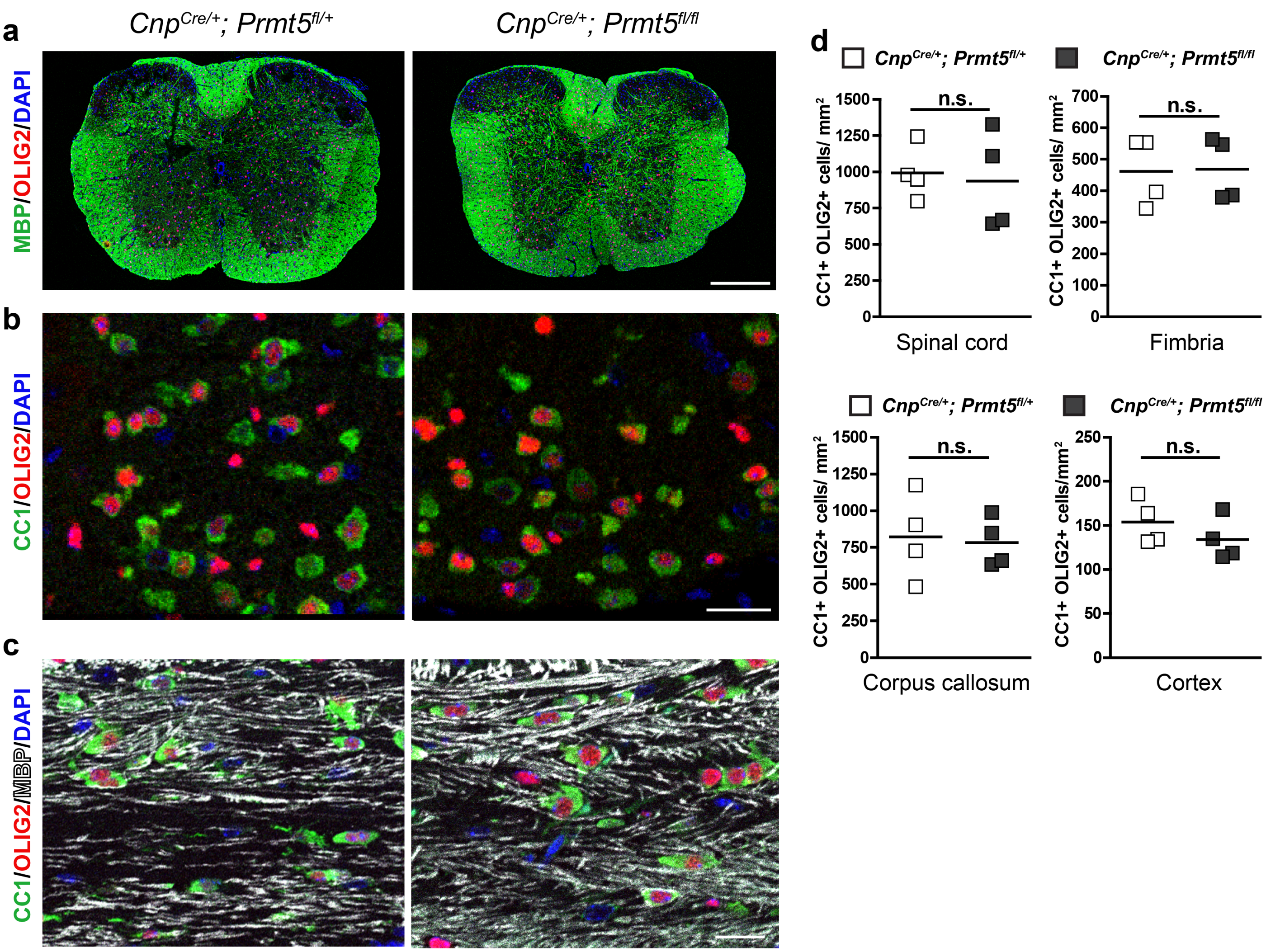


**Supplementary Figure 3. Genetic ablation of *Prmt5* in oligodendrocytes using *Cnp-Cre*.** Confocal images of P14 spinal cord (**a**,**b**) sections from controls *($Olig1^{Cnp/+}$;$Prmt5^{fl/+}$)* and *Prmt5* mutants (*$Olig1^{Cnp/+}$;$Prmt5^{fl/fl}$*) stained for (**a**) MBP (green), (**b**) CC1 (green), (**a**, **b**) OLIG2 (red) and DAPI (blue). Scale bar in **a**: 200µm , in **b**: 20µm. (**c**) Confocal images of P14 corpus callosum sections from controls *($Olig1^{Cnp/+}$;$Prmt5^{fl/+}$)* and *Prmt5* mutants (*$Olig1^{Cnp/+}$;$Prmt5^{fl/fl}$)* stained for MBP (white), CC1 (green), OLIG2 (red) and DAPI as nuclear counterstain (blue). Scale bar: 20µm. (**d**) Scatter plot represents the average number of $CC1^{+}$ /$OLIG2^{+}$ cells quantified in in spinal cord, fimbria, corpus callosum, and cortex from four controls *($Olig1^{Cnp/+}$;$Prmt5^{fl/+}$)* and four *Prmt5* mutants (*$Olig1^{Cnp/+}$;$Prmt5^{fl/fl}$).* Student's *t*-test, n.s.= not significant.

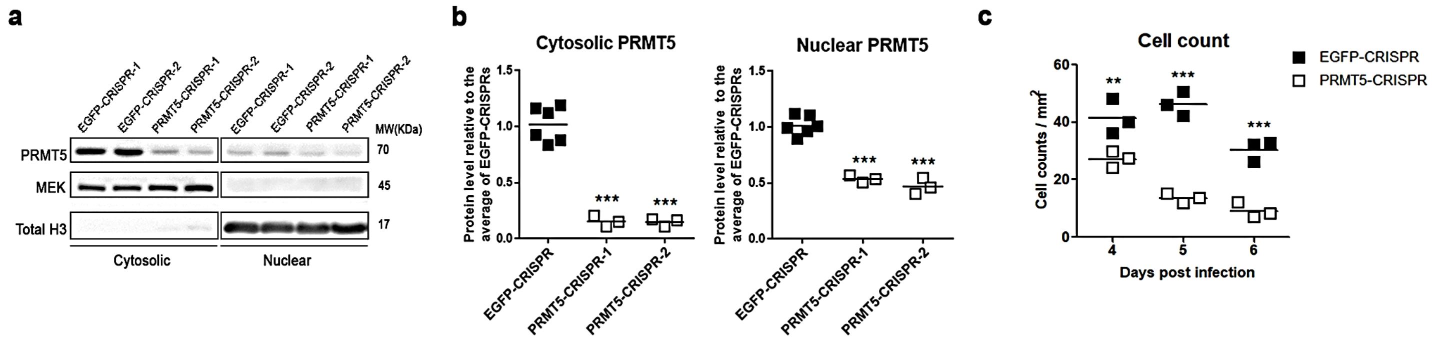


**Supplementary Figure 4. Validation of PRMT5 knockdown in oligodendrocytes lineage cells, using CRISPR/Cas9.** (**a**) Western blot analysis of cytosolic or nuclear protein extracts from OPC either infected with the control (EGFP-CRISPR) or knockdown (PRMT5-CRISPR) lentiviruses and probed with antibodies for PRMT5, MEK was used as control for cytoplasmic proteins and total H3 as control for nuclear proteins. (**b**) Relative quantification of the PRMT5 protein level in control (EGFP-CRISPR) or knockdown (PRMT5-CRISPR) cytosolic and nuclear extracts is shown. Data were normalized to the appropriate loading controls and are presented as means from three different preparations (one-way ANOVA with Bonferroni's Multiple Comparison Test, *** $p<0.001$). (**c**) Absolute numbers of OPC cells infected with either control (EGFP-CRISPR) or knockdown (PRMT5-CRISPR) lentiviruses, at day 4, 5 and 6 after infection. Scatter plots indicate relative values normalized to the average of the control group (DMSO), (one-way ANOVA, ** $p<0.01$, *** $p<0.001$), n=3 independent preparations.

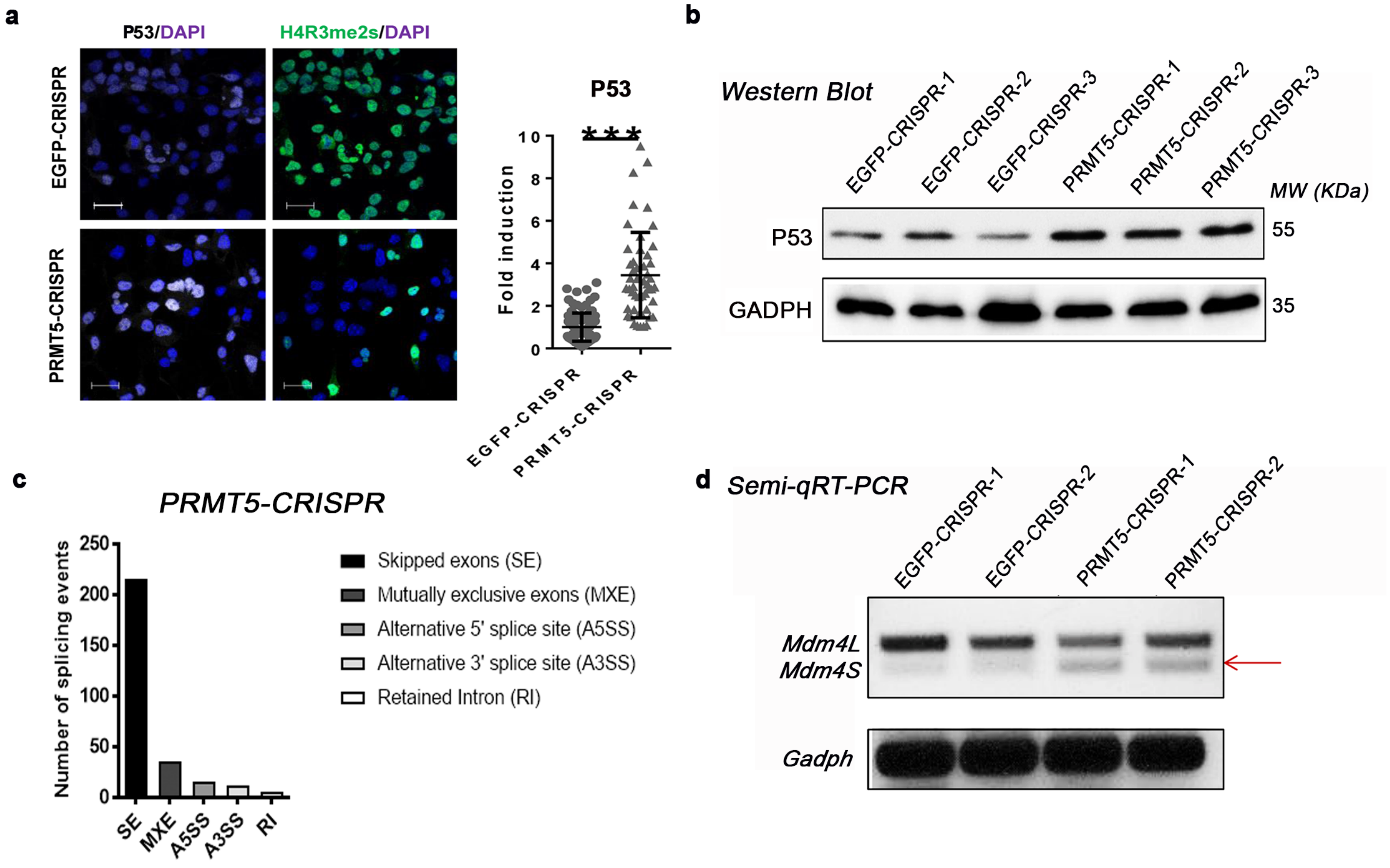


**Supplementary Figure 5. CRISPR/Cas9 targeting of PRMT5 in oligodendrocyte progenitors leads to alteration of *Mdm4* splicing and up-regulation of P53 protein levels.** (**a**) Representative confocal images of oligodendrocyte progenitor cells with (PRMT5-CRISPR) or without (EGFP-CRISPR) targeting of PRMT5 using lentiviral vectors, processed for immunocytochemistry using antibodies specific for P53 (gray) and for H4R3me2s (green). DAPI (blue) was used as nuclei counter-stain. Scale: 20 μm. Scatter plot identify the intensity of p53 immunoreactivity in H4R3me2s$^{+}$ (EGF-CRISPR) and in H4R3me2s$^{-}$ (PRMT5-CRISPR) cells. Data are shown as normalized values, relative to the average of the EGFP group ± s.e.m. from three independent preparations. *** =p<0.001 Student's *t*-test. (**b**) Whole cell protein lysates extracted from progenitors transduced with control (EGFP-CRISPR) or targeted (PRMT5-CRISPR) lentiviral vectors were processed for Western blot analysis and probed with antibodies specific for P53 and for GAPDH, as loading control. (**c**) Relative distribution of RNA splicing events detected in Olineu cells after PRMT5 knockdown (PRMT5-CRISPR). (**d**) Semi-qRT-PCR detecting the transcripts of the *Mdm4* long and short isoforms in EGFP-CRISPR or PRMT5-CRISPR infected oligodendrocyte progenitors.

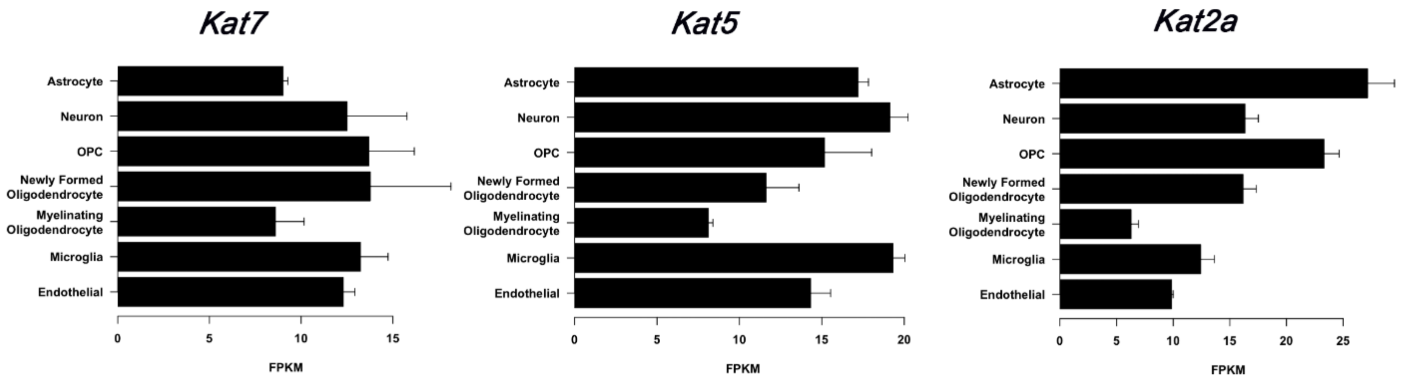


**Supplementary Figure 6. Transcript levels of acetyltransferase from available databases**. The transcript values of *Kat7*, *Kat5* and *Kat2a* in different cell classes of the brain as reported in RNA-seq transcriptome database[1,2]. Note the enrichment of the three transcripts in the OPC.

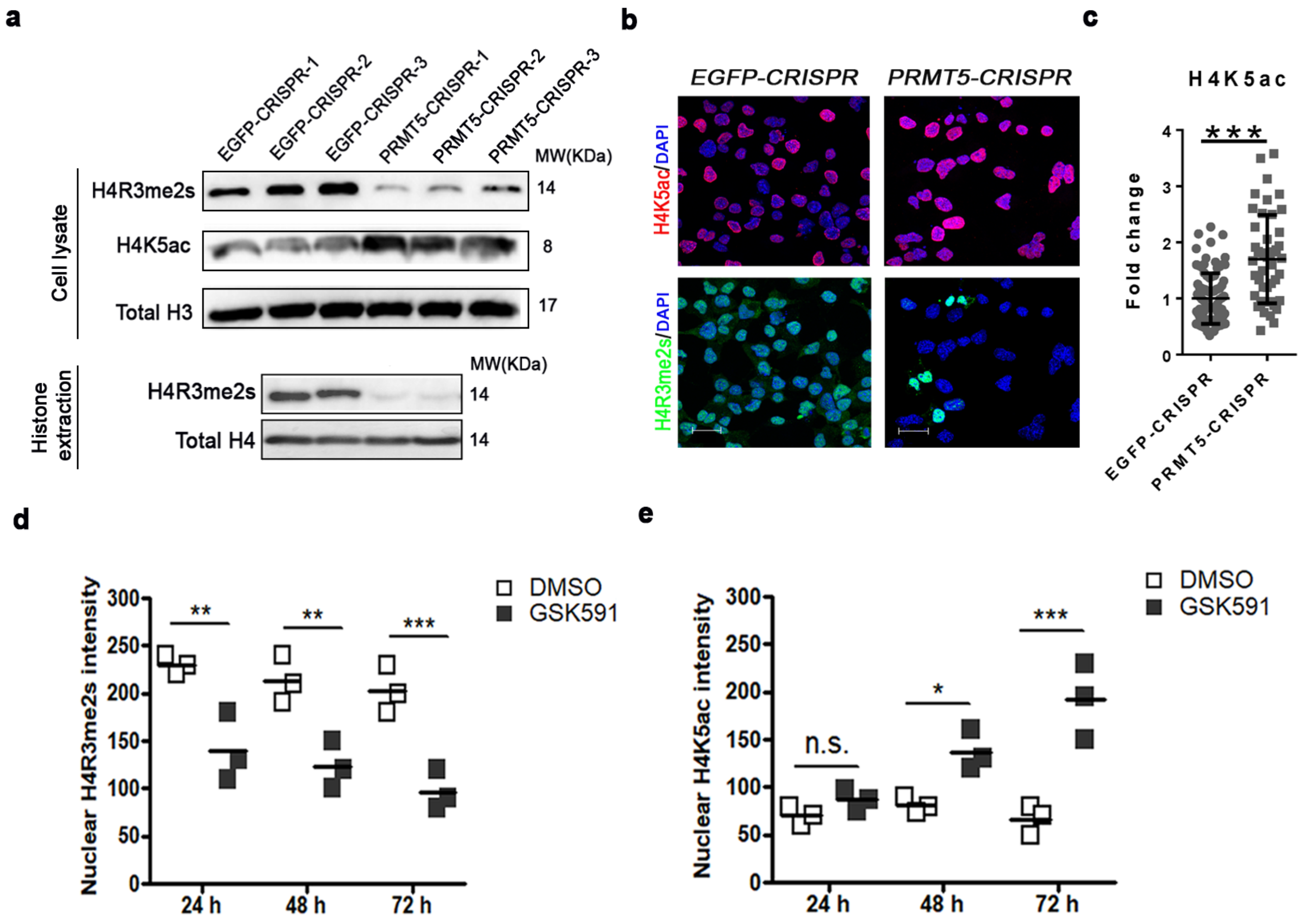


**Supplementary Figure 7. Increased acetylation of lysine residue K5 in histone H4 after PRMT5 knockdown.** (**a**) Western blot analysis of cell lysates and histone extraction from control (EGFP-CRISPR) and knockdown (PRMT5-CRISPR) cultures probed with antibodies specific for H4R3me2s and for H4K5ac. Antibdes for total H3 or total H4 were used as protein loading control for histones. (**b**) Representative confocal images of control (EGFP-CRISPR) and knockdown (PRMT5-CRISPR) OliNeu cells stained with antibodies specific for H4K5ac (red), H4R3me2s (green) and DAPI (blue). (**c**) Scatter plot of the normalized immunoreactivity intensity for H4K5ac staining counted in at least 50 cells per condition and from three independent biological replicates. Scatter plots indicate relative values normalized to the average of the EGFP group ± s.e.m. Student's t-test, *** p<0.001, scale 20 μm. (**d-e**) Time course of the effect of the pharmacological inhibitor of PRMT5 (GSK591, 10 nM) treatment on methyl-arginine (H4R3me2s) and acetyl-lysine (H4K5ac) deposition in primary oligodendrocyte progenitors, cultured in presence of growth factors (+GF), for 24 and 72 hours. Scatter plot of the average immunoreactivity intensity of nuclear H4R3me2s and H4K5ac staining. Each dot represents the average of 50 PDGFR$\alpha^{+}$ cells from three independent preparations. The values werenormalized to the average of the control group (DMSO). One-way ANOVA n.s. p>0.05 * p<0.05, ** p<0.01, *** p<0.001.

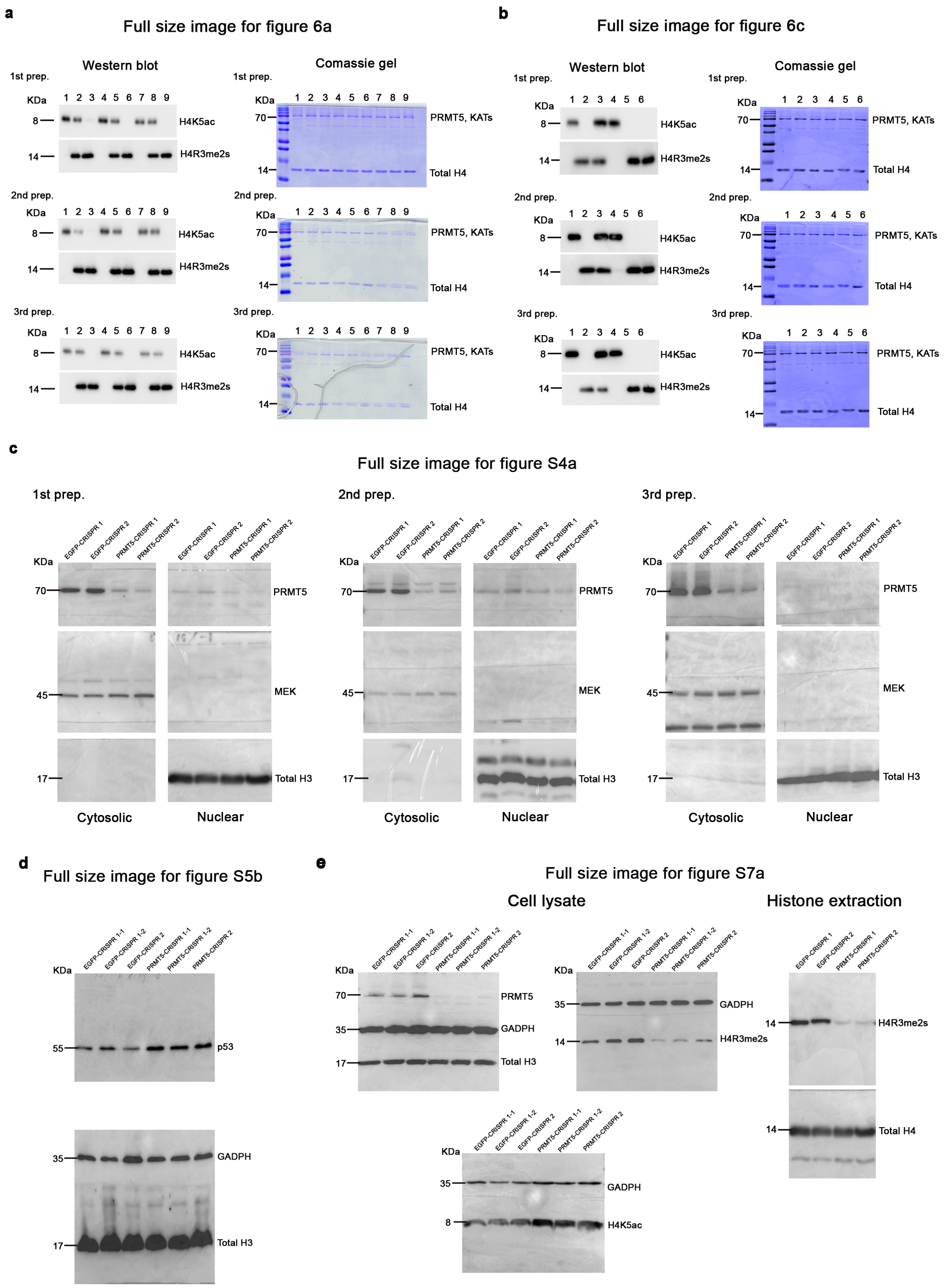


**Supplementary Figure 8. Original western blots of data presented in this manuscript**. Uncropped images of immunoblots and coomassie gels displayed in the main and supplementary figures. Antibodies indicated on the right side and size markers are indicated on the left side of each blot.

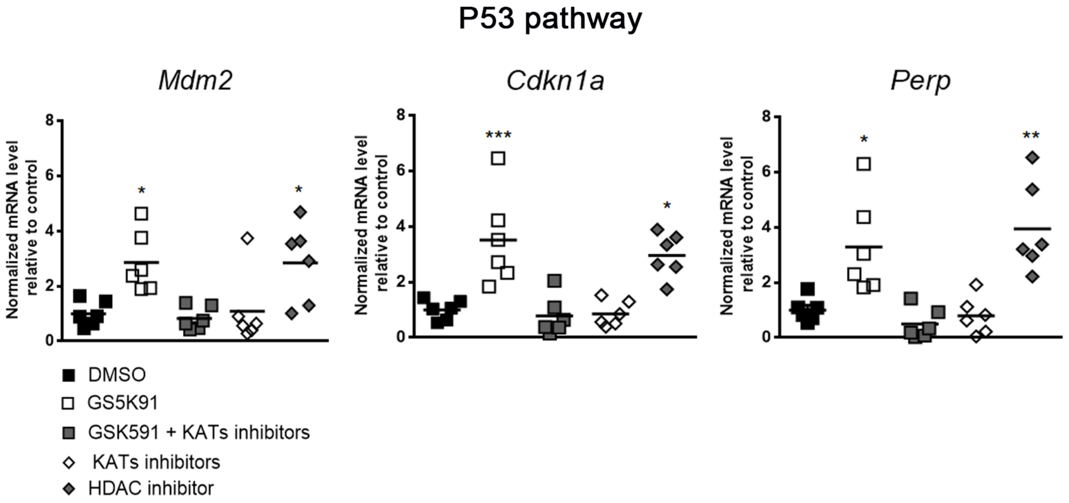


**Supplementary Figure 9. Upregulation of P53-dependent genes induced by PRMT5 inhibition is rescued by co-treatment with KAT inhibitors.** qRT-PCR of selected P53-target genes (*Mdm2, Cdkn1a, Perp*) normalized to the geo-mean of three housekeeping genes (*18s*, *Wdr33* and *Pja2*). Differentiating oligodendrocyte progenitors were treated for 48 hours with DMSO, KAT inhibitors (Butyrolactone-3, 100 μM and Nu-9056, 0.2 μM) alone or in combination with the PRMT5 inhibitor GSK591 (10nM) or with the HDAC inhibitor Trichostatin A (TSA, 20 nM). Note that TSA is toxic and induces upregulation while the KAT inhibitors rescue the upregulation induced by GSK591. Scatter plots indicate transcript values for the indicated genes from six independent preparations. One-way ANOVA with Bonferroni Multiple Comparison Test was performed for each gene compared to the value of the control (DMSO) (* p<0.05** p<0.01 *** p<0.001).

## Supplementary Tables

**Supplementary Table 1.** List of primary antibodies used in the study.

| Technique | Antibody name | Antibody source | Commercial house and reference | Dilution |
|---|---|---|---|---|
| Immunopanning | CD140a | Rat | BD Bioscience 558774 | 1:1000 |
| Immunoprecipitation | PRMT5 | Rb | Abcam, ab31751 | |
| Histone peptide array (Active Motif, 13005) | H3R2me2s | Rb | Ernesto Guccione's | |
| | H4R3me2a | Rb | Active Motif, 39705 | |
| | H4R3me2s | Rb | Abcam, ab5823 | |
| | H4R3me2s | Rb | Epigentek, A-3718 | |
| Western Blot | GADPH | Ms | Abcam, ab8245 | 1:5000 |
| | H3 | Rb | Abcam ab1791 | 1:20000 |
| | H3K9ac | Ms | Active Motif, 61251 | 1:1000 |
| | H4 | Rb | Active motif, 39269 | 1:1000 |
| | H4R3me2s | Rb | Epigentek A-3718 | 1:1000 |
| | H4K5ac | Ms | Active Motif, 61523 | 1:1000 |
| | H4K5ac | Rb | Abcam, ab51997 | 1:1000 |
| | H4K8ac | Rb | Millipore, 07-328 | 1:1000 |
| | H4K12ac | Rb | Millipore, 07-595 | 1:1000 |
| | MEK | Rb | Sigma, M 5795 | 1:5000 |
| | PRMT5 | Rb | Abcam, ab109451 | 1:1000 |
| Immunohistochemistry | CC1 | Ms | Calbiochem, OP80 | 1:100 |
| | H4R3me2s | Rb | Diagenode, C15410308 | 1:200 |
| | H4K5Ac | Ms | Active Motif, 61523 | 1:200 |
| | Ki67 | Rb | Abcam, ab15580 | 1:500 |
| | MBP | Rat | Millipore MAB386 | 1:500 |
| | Olig2 | Rb | Chemicon, AB9610 | 1:200 |
| | Olig2 | Ms | Millipore, MABN50 | 1:200 |
| | PDGFR$\alpha$ | Rb | Cell Signalling, 3164S | 1:100 |
| | PRMT5 | Rb | Abcam, AB109451 | 1:100 |

| | | | | |
|---|---|---|---|---|
| Immunocytochemistry | Cleaved CASPASE-3 | Rb | Cell Signalling, 9661 | 1:200 |
| | CNP | Ms | Covance, E11BF00277 | 1:200 |
| | H4R3me2s | Rb | Diagenode, C15410308 | 1:500 |
| | H4K5Ac | Ms | Active Motif, 61523 | 1:500 |
| | Ki67 | Ms | Abcam, ab15580 | 1:500 |
| | MBP | Rat | Millipore MAB386 | 1:1000 |
| | Olig2 | Rb | Chemicon, AB9610 | 1:500 |
| | Olig2 | Ms | Millipore, MABN50 | 1:500 |
| | P53 | Rb | Cell Signalling, 2524S | 1:500 |
| | PRMT5 | Rb | Abcam, AB109451 | 1:200 |
| | PDGFR$\alpha$ | Rat | Millipore, CBL1366 | 1:1000 |

**Supplementary Table 2.** Number of total reads and aligned read pairs for the RNA-seq analysis performed for CRISPR-PRMT5 and CRISPR-EGFP control cells. The table shows the values obtained from four different analysis performed for each condition (CRISPR-PRMT5 and CRISPR-EGFP control)

| Sample | Total read pairs | Aligned read pairs | Average of total read pairs | Range of total read pairs | Read type |
|---|---|---|---|---|---|
| EGFP-CRISPR-1 (exp.1) | 64722813 | 52660045 | 61602313.25 | 56743181 - 66929862 | Paired end |
| EGFP-CRISPR-2 (exp. 1) | 66929862 | 54600276 | | | Paired end |
| EGFP-CRISPR-1 (exp.3) | 56743181 | 45668535 | | | Paired end |
| EGFP-CRISPR-2 (exp.4) | 58013397 | 47106242 | | | Paired end |
| PRMT5-CRISPR-3 (exp. 1) | 68576976 | 55956459 | 63379315.75 | 59009841 - 68576976 | Paired end |
| PRMT5-CRISPR-4 (exp. 2) | 64201644 | 52395588 | | | Paired end |
| PRMT5-CRISPR-3 (exp. 3) | 59009841 | 47970026 | | | Paired end |
| PRMT5-CRISPR-4 (exp. 4) | 61728802 | 49934101 | | | Paired end |

**Supplementary Table 3.** List of qRT-PCR primers used in the study

| Gene | Forward primer | Reverse primer |
|---|---|---|
| *Prmt5* | CTGAGTGTCTGGATGGAGCA | GCATCTCAAACTGTGCCTCA |
| *Cdkn1a* | CAGGGCAGAGGAAGTACTGG | CGGTGGAACTTTGACTTCGT |
| *Mdm2* | TAAAGTCCGTTGGAGCGCAAA | CTGCTGCTTCTCGTCATATAACC |
| *Perp* | ACCCCAGATGCTTGTTTTCCT | CTGCTGCTTCTCGTCATATAACC |
| *Gpr17* | ACACATTGTCTGCCTGCAA | TGACCGTGGTGATGAATGGG |
| *Myt1* | TGCAGACCTCAGTTGTCCTAC | TCCTCTTGGATACCAGGTGCT |
| *Sox10* | GGAGATCAGCCACGAGGTAATG | GTTGGGTGGCAGGTATTGGT |
| *Cnp* | ACCCGCAAAAGCCACACA | CACCGTGTCCTCATCTTGAAG |
| *Mbp* | ACACACGAGAACTACCCATTATGG | AGAAATGGACTACTGGGTTTTCATCT |
| *m18s* | AGTCCCTGCCCTTTGTACACA | GATCCGAGGGCCTCACTAAAC |
| *Pja2* | GCC TTG CCA TCA CTT CTT TC | GCA GAT GCG TCA ATA ACT GC |
| *Wdr33* | TGA TCT GGT CCC ACC AAT AG | GCA GAT GCG TCA ATA ACT GC |

**Supplementary Table 4.** List of the genes identified in PRMT5 knockdown cells compared to controls for the following alternative splicing event: Skipped Exon (SE), Mutually Exclusive Exon (MXE), Alternative 5' splice event (A5SS), Alternative 3' splice event (A3SS), Retained Intron (RI)

| SE | MXE | A5SS | A3SS | RI |
|---|---|---|---|---|
| *Myo5a* | *Rsbn1l* | *Zfp691* | *Zfp2* | *Hsf1* |
| *Supl15h* | *Sobp* | *Sh3bp5l* | *Epn1* | *Adamts10* |
| *Prpf39* | *Vit* | *Adamts10* | *Map4k4* | *Pla2g4b* |
| *Crem* | *Zfp346* | *Steap2* | *Tbc1d24* | *Amigo2* |
| *Uspl1* | *Ints10* | *Taf1c* | *Fkbp1a* | *Keap1* |
| *Aste1* | *Fuk* | *Lzts3* | *Zfp788* | *Tert* |
| *Csnk1g3* | *Tmbim6* | *Ciz1* | *Zbtb37* | |
| *Sbno2* | *Dap3* | *Ccser1* | *Zfp946* | |
| *Pdp1* | *Klhl18* | *Rfx5* | *Baz2b* | |
| *Sobp* | *Ptk2* | *Il15ra* | *Tank* | |
| *Map3k4* | *Zfp788* | *Adamts10* | *Pms2* | |
| *Atp5s* | *Enox2* | *Sema6c* | *Zfp82* | |
| *Pkd1* | *Zfp788* | *Apoo* | | |
| *Atr* | *A730011L01Rik* | *Rsad1* | | |
| *edpk* | *9530077C05Rik* | *Cant1* | | |
| *AK016788* | *Tcf7l2* | | | |
| *B3gnt5* | *Pdgfc* | | | |
| *Eps15l1* | *Epb41l4a* | | | |
| *Enox2* | *Smarce1* | | | |
| *Armcx1* | *Sorbs1* | | | |
| *Trim68* | *Ap4e1* | | | |
| *Nsun3* | *Prss36* | | | |
| *Zfp788* | *Senp8* | | | |
| *Ankrd29* | *Lrrc7* | | | |
| *Fbrsl1* | *Kif21a* | | | |

| | | | | |
|---|---|---|---|---|
| *mKIAA1584* | *Cyp2r1* | | | |
| *Kirrel3* | *Scyl3* | | | |
| *1110054O05Rik* | *Lrrc7* | | | |
| *BC006965* | *Dnahc9* | | | |
| *2310005N01Rik* | *COX4AL* | | | |
| *Tmbim6* | *Senp8* | | | |
| *Mllt4* | *Dnahc9* | | | |
| *Sdccag3* | *Cnot2* | | | |
| *Krba1* | *Enox2* | | | |
| *mKIAA1335* | *Kif21a* | | | |
| *Phf16* | | | | |
| *Svil* | | | | |
| *Fam151b* | | | | |
| *Enox2* | | | | |
| *Zfp202* | | | | |
| *Dos* | | | | |
| *Zfp821* | | | | |
| *Cep152* | | | | |
| *Krba1* | | | | |
| *Ocel1* | | | | |
| *Acvr1* | | | | |
| *Tmtc1* | | | | |
| *Zfyve21* | | | | |
| *Senp8* | | | | |
| *Gal3st4* | | | | |
| *Mylk* | | | | |
| *Senp8* | | | | |
| *Vegfa* | | | | |

| | | | | |
|---|---|---|---|---|
| *Wdr52* | | | | |
| *Acap2* | | | | |
| *Trerf1* | | | | |
| *Zfp788* | | | | |
| *Ccdc77* | | | | |
| *Abca8b* | | | | |
| *Pla2g4b* | | | | |
| *Zfp324* | | | | |
| *4930578N16Rik* | | | | |
| *Zfp239* | | | | |
| *St3gal3* | | | | |
| *Cacna1c* | | | | |
| *AK015657* | | | | |
| *Slc7a3* | | | | |
| *Pbx4* | | | | |
| *Rgs19* | | | | |
| *4933411K20Rik* | | | | |
| *Tmem194b* | | | | |
| *Cyp2r1* | | | | |
| *Osgepl1* | | | | |
| *Tns1* | | | | |
| *Elmod3* | | | | |
| *Itgav* | | | | |
| *4932438A13Rik* | | | | |
| *Ppp4r1l-ps* | | | | |
| *Nfatc2ip* | | | | |
| *Zfp324* | | | | |
| *Alkbh1* | | | | |

| | | | | |
|---|---|---|---|---|
| *Baz2b* | | | | |
| *Slmap* | | | | |
| *Wdr62* | | | | |
| *6720401G13Rik* | | | | |
| *Atg16l1* | | | | |
| *Atp4a* | | | | |
| *Lrrc45* | | | | |
| *Brd4* | | | | |
| *1810013D10Rik* | | | | |
| *Clta* | | | | |
| *Sgip1* | | | | |
| *4930523C07Rik* | | | | |
| *Wnk1* | | | | |
| *Accs* | | | | |
| *mKIAA0656* | | | | |
| *0610037D15Rik* | | | | |
| *Erc2* | | | | |
| *Polm* | | | | |
| *BC046404* | | | | |
| *Agbl2* | | | | |
| *Zfp655* | | | | |
| *2010015L04Rik* | | | | |
| *Tcf7l2* | | | | |
| *6230409E13Rik* | | | | |
| *Anubl1* | | | | |
| *Cyp2r1* | | | | |
| *Ddb2* | | | | |
| *Sgip1* | | | | |

| | | | | |
|---|---|---|---|---|
| *Baz2b* | | | | |
| *2310004I24Rik* | | | | |
| *Ubap2l* | | | | |
| *Armcx1* | | | | |
| *P2rx6* | | | | |
| *Znf618* | | | | |
| *Pigv* | | | | |
| *Fam38b* | | | | |
| *Nkain4* | | | | |
| *Terf2* | | | | |
| *Odz4* | | | | |
| *4930481A15Rik* | | | | |
| *Ccdc157* | | | | |
| *Neil1* | | | | |
| *Fggy* | | | | |
| *meif2C4* | | | | |
| *AK045786* | | | | |
| *Csmd1* | | | | |
| *Ttc13* | | | | |
| *Dnajc17* | | | | |
| *Zfp952* | | | | |
| *Wipf3* | | | | |
| *Tcf20* | | | | |
| *Cyp2r1* | | | | |
| *Gprasp2* | | | | |
| *2210009G21Rik* | | | | |
| *Gbp3* | | | | |
| *Ccdc57* | | | | |

| | | | | |
|---|---|---|---|---|
| *Ptk2* | | | | |
| *Cep63* | | | | |
| *Prss36* | | | | |
| *Gprasp2* | | | | |
| *Sh3glb2* | | | | |
| *BC004004* | | | | |
| *Rgs7* | | | | |
| *Rcbtb1* | | | | |
| *Ranbp17* | | | | |
| *Fbxo25* | | | | |
| *Grhl1* | | | | |
| *Tec* | | | | |
| *Mbd5* | | | | |
| *Tmem194b* | | | | |
| *Gpr137* | | | | |
| *Poli* | | | | |
| *Pion* | | | | |
| *Mark3* | | | | |
| *Scn2a1* | | | | |
| *Cyp4f17* | | | | |
| *Wdr78* | | | | |
| *AI118078* | | | | |
| *Abcb4* | | | | |
| *Slc39a14* | | | | |
| *Lrp8* | | | | |
| *Zbtb43* | | | | |
| *Slc17a8* | | | | |
| *Slc29a3* | | | | |

| | | | | |
|---|---|---|---|---|
| *Tgfbr2* | | | | |
| *Cd99l2* | | | | |
| *Ppm1b* | | | | |
| *Raph1* | | | | |
| *Prpf40b* | | | | |
| *2210404J11Rik* | | | | |
| *Zfp551, Znf551* | | | | |
| *Fggy* | | | | |
| *Pdzd2* | | | | |
| *2810429I04Rik* | | | | |
| *Gfra1* | | | | |
| *Ccdc76* | | | | |
| *Tsku* | | | | |
| *Zbtb43* | | | | |
| *Shc1* | | | | |
| *Crem* | | | | |
| *Madd* | | | | |
| *Lipo1* | | | | |
| *Rapgef6* | | | | |
| *Rims1* | | | | |
| *Dsc2* | | | | |
| *Enox2* | | | | |
| *6720401G13Rik* | | | | |
| *Lrp1b* | | | | |
| *Thap6* | | | | |
| *Wdfy2* | | | | |
| *4930432K21Rik* | | | | |
| *Thsd1* | | | | |

| | | | | |
|---|---|---|---|---|
| *E130303B06Rik* | | | | |
| *Fam35a* | | | | |
| *Kif24* | | | | |
| *Blzf1* | | | | |
| *Zfp317* | | | | |
| *Rapgefl1* | | | | |
| *Tpd52l1* | | | | |
| *D2hgdh* | | | | |
| *Nr3c2* | | | | |
| *Sytl4* | | | | |
| *Scly* | | | | |
| *Trpm2* | | | | |
| *Pgbd1* | | | | |
| *Dlg3* | | | | |
| *1700012B15Rik* | | | | |
| *Zfp420* | | | | |
| *Xrcc3* | | | | |
| *Tpcn2* | | | | |
| *2210009G21Rik* | | | | |
| *Thap6* | | | | |
| *Slc35a1* | | | | |
| *Mfsd9* | | | | |

## Supplementary References


1. Zhang *et al.,* An RNA-Sequencing Transcriptome and Splicing Database of Glia, Neurons, and Vascular Cells of the Cerebral Cortex, *Journal of Neuroscience*, **34** (36) 11929-11947; DOI: https://doi.org/10.1523/JNEUROSCI.1860-14 (2014).
2. http://www.brainrnaseq.org
