# Supplementary material for: PRMT5-mediated regulation of developmental myelination
Source: Nat Commun. 2018 Jul 19;9:2840. doi: 10.1038/s41467-018-04863-9 (PMC6053423; doi:10.1038/s41467-018-04863-9)
Supplement: Supplementary file 2 — Description of Additional Supplementary Files [file 41467_2018_4863_MOESM2_ESM.pdf]

Description of Additional Supplementary Files

File Name: Supplementary Data 1
Description: List of most significantly differentially regulated transcripts after loss of Prmt5.
